# *Hypericum sampsonii* Hance: a review of its botany, traditional uses, phytochemistry, biological activity, and safety

**DOI:** 10.3389/fphar.2023.1247675

**Published:** 2023-09-19

**Authors:** Zhanghua Sun, Yanzhen Li, Ruimin Zhong, Ran Li

**Affiliations:** ^1^ Guangdong Provincial Key Laboratory of Utilization and Conservation of Food and Medicinal Resources in Northern Region, Shaoguan University, Shaoguan, China; ^2^ College of Food Science and Technology, Shaoguan University, Shaoguan, China; ^3^ School of Pharmaceutical Sciences, Guangzhou University of Chinese Medicine, Guangzhou, China

**Keywords:** *Hypericum sampsonii* Hance, botany, traditional uses, phytochemistry, biological activities, safety

## Abstract

**Ethnopharmacological relevance:** Hypericum sampsonii Hance, also known as Yuanbao Cao in Chinese, is a traditional medicinal herb from the Guttiferae family and has been widely used in China to treat various conditions, including dysentery, enteritis, mastitis, scrofula, and contusion.

**Aim of the review:** This review aims to provide a comprehensive overview of the botany, traditional uses, phytochemistry, biological activity and safety of *H. sampsonii* and to highlight its potential for medical application and drug development.

**Materials and methods:** We searched several databases, i.e., Web of Science, SciFinder, PubMed, CBM, CNKI, Google Scholar, etc., for relevant information on *H. sampsonii*. Additionally, we also consulted some books on Chinese medicine.

**Results:** To date, 227 secondary metabolites have been isolated from *H. sampsonii*, including polycyclic polyprenylated acylphloroglucinols (PPAPs), benzophenones, xanthones, flavonoids, naphthodianthrones, anthraquinones and aromatic compounds. These metabolites exhibit various biological activities such as anti-inflammatory, anti-tumor, anti-depressant, anti-oxidant, anti-viral and anti-bacterial effects. PPAPs are considered the main active metabolites with rich biological activities. Despite being known as rich source of PPAPs, the full extent of *H. sampsonii* biological activities, including their potential as PDE4 inhibitors, remained unclear. Since, previous studies have mainly been based on structural identification of metabolites in *H. sampsonii*, and efficacy evaluations of these metabolites based on clinical applications of *H. sampsonii* lack sufficient data. However, current evidence suggest that PPAPs are the most likely material basis for efficacy. From the limited information available so far, there is no evidence of potential safety issues and the safety data are limited.

**Conclusion:** Collectively, this review provides a comprehensive overview of the botany, traditional uses, phytochemistry, pharmacology, and safety of *H. sampsonii*, a valuable medicinal plant in China with various pharmacological activities. Based on pharmacological studies, *H. sampsonii* shows potential for treating gastrointestinal and gynecological disorders as well as traumatic injuries, which aligns with traditional medicinal use due to the presence of PPAPs, benzophenones, xanthones, and flavonoids. Therefore, further studies are needed to evaluate the pharmacological effects and elucidate the pharmacological mechanisms. In addition, pharmacological mechanisms and safety evaluation of PPAPs on animal models need to be clarified. Yet, further comprehensive studies are required to elucidate the phytochemical constituents, pharmacological mechanisms, structure-activity relationships, safety evaluation, and quality standards of this plant. Takentogether, this review highlights the potential of *H. sampsonii* for medical application and drug development.

## 1 Introduction

Plants have been used in traditional medicine for centuries to prevent and treat various diseases. Ethnomedicinal plants, which have clinical evidence of efficacy and safety, play an important role in drug discovery and development (Cragg and Pezzuto, 2016; Anand et al., 2019; Choudhari et al., 2020). The *Hypericum* genus (Guttiferae) boasts over 460 species that are distributed worldwide, with the exception of arctic and desert regions and most tropical lowlands. (Committee, 2007). Some species are widely used in official medicine throughout the world, such as *Hypericum perforatum* L. (St. John’s wort) (Stojanovic et al., 2013). However, some endemic species of *Hypericum* have traditionally been used as folk medicine or ethnomedicine in East Asia, particularly in China (Zhang et al., 2020). *Hypericum sampsonii* Hance (known as “Yuanbao Cao” in Chinese), which is used as a traditional medicine in south of Changjiang River, has been commonly used as a folk medicine with functions of traumatic bleeding, enteritis, dysentery, and acute mastitis (Gong, 2014; Xie et al., 2021).

Recent years have shown, growing interest of researchers in the chemical constituents and pharmacological effects of *H. sampsonii*. Previous chemical investigation of *H*. *sampsonii* reports a series of metabolites including polycyclic polyprenylated acylphloroglucinols (PPAPs), benzophenones, flavonoids, xanthones, naphthodianthrones, anthraquinones, and aromatic compounds (Xie et al., 2021). The previously reported literatures have revealed that *H*. *sampsonii* possesses multiple biological properties, including anti-inflammatory, antinociceptive, antitumor, antidepressant, antimicrobial, antiviral, and antioxidant activities (Tian, 2015; Xie et al., 2021). Accordingly, in the present srudy, we have attempted a pharmacological analysis of the whole plant *H. sampsonii* to understand the primary target of inflammation and to validate the ethnomedicine reports due to its numerous pharmacological activities and traditional claims of anti-inflammatory properties in the intestinal tract (Lin et al., 2022). However, biological activities and molecular mechanisms of constituents in *H*. *sampsonii* have not been fully explored, and a comprehensive and systematic review of this plant is lacking. The present study will not only provide motivation to the growing interest in recent years for a better understanding of the indication-discovery strategies but also assist the concept of drug repurposing in the treatment of many other related clinical conditions that may direct guide towords future research plan.

In this review, the botany, traditional uses, phytochemistry, pharmacological action, and safety of *H*. *sampsonii* have been summarized along with discussion over future direction and focus of *H*. *sampsonii* in the field of pharmacology.

## 2 Materials and methods

The relevant information was collected from various search engines: Web of Science, SciFinder, PubMed, CBM, CNKI, Google Scholar, etc. Other literature sources, i.e., classic books of Chinese herbal medicine were also screened to get the maximal information on this plant. The keywords used included *H. sampsonii* Hance, botany, phytochemistry, pharmacological activity, traditional uses, safety, and other related words. The plant name was also checked with World Flora Online (WFO (2023): Hypericum sampsonii Hance. Published on the Internet; http://www.worldfloraonline.org/taxon/wfo-0000728267. Accessed on: 04 February 2023).

## 3 Botany

**FIGURE 1:**
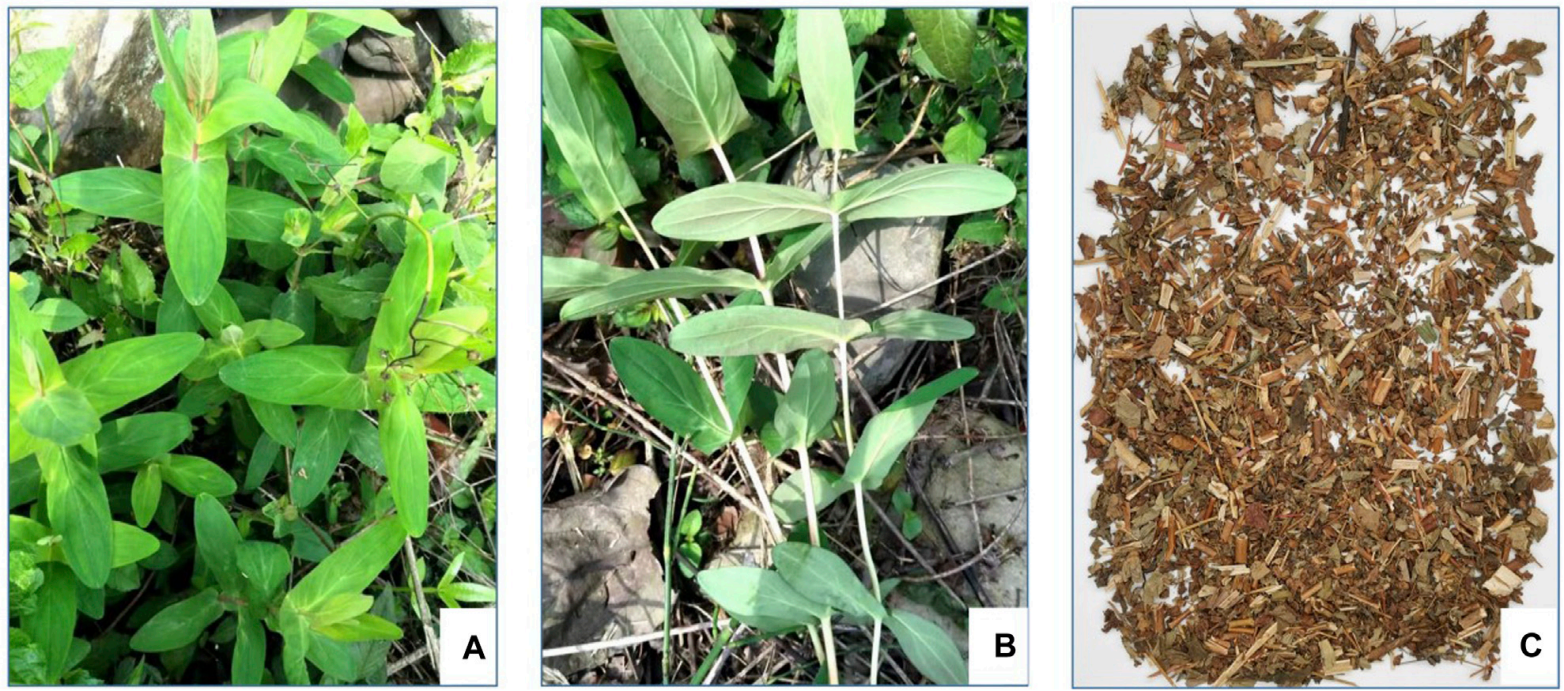
Photographs of *H. sampsonii*: the whole plant **(A)**, the lateral view of connate perfoliate leaves **(B)**, and medicinal material **(C)**.

According to World Flora Online, this name of *H. sampsonii* Hance (Figure 1) of Hypericaceae family has been accepted, with other four synonyms including *Hypericum electrocarpum* Maxim, *H. electrocarpum* f. *parvifolium* R. Keller, *Hypericum esquirolii* H. Lév, and *Hypericum oshimaense* R. Keller, in the genus *Hypericum* (family Hypericaceae). As a folk medicine, various vernacular names of *H*. *sampsonii* have been known in China, such as Hezhang Cao, Shangtianti, Dahuanhun, etc (Table 1).

**TABLE 1:** Vernacular names of *H. sampsonii* in China.

Area	Name	Ref.
Jiangxi	Xiangsi, Dengtai, Shuanghehe, Duiye Cao, Daduiye Cao, Pai Cao, Duijing Cao, Sanxuedan	Wu, 1963; Zhang et al. (2020)
Guangxi	Daduiye Cao, Yebaozhi, Fanchuan Cao, Xiaohuangxin Cao, Baoxin Cao, Waxin Cao, Yangxingai (Miao people), Shanglianxialiu (Zhuang people), Mawu (Mulam people)	Wu, 1991; Zhang et al. (2020)
Fujian	Xiangsi, Dengtai, Shuanghehe, Duiye Cao, Yebaozhi, Dangguilian, Xiaolianqiao, Duilian, Ligenxiang, Danggui Cao	Zhang et al. (2020)
Zhejiang	Baota Cao, Chuanxinjian, Lazhudengtai, Dengtai, Qingting Cao, Honghanlian, Dayeyeyanzi	Wu, 1963; Zhang et al. (2020)
Guizhou	Xiangsi, Dengtai, Shuanghehe, Duiye Cao, Duiyuelian, Shezhakou	Zhang et al. (2020)
Hunan	Daduiye Cao, Wangbuliuxing, Liujinu, Shaoxi Cao, Lanchang Cao, Yehanyan, Mangzi Cao, Shekaikou, Yangzi Cao, Yizi Cao, Jiaozhu Cao	Zhang et al. (2020)
		*Hunan Yaowu Zhi*
		《湖南药物志》
Hubei	Daduiye Cao, Wangbuliuxing, Liujinu, Duiyue Cao	Zhang et al. (2020)
Sichuan	Daduiye Cao, Foxin Cao, Duidui Cao	Wu, 1963; Zhang et al. (2020)
Jiangsu	Kongxin Cao, chuanxin Cao	Zhang et al. (2020)
Guangdong	Daduiye Cao	Zhang et al. (2020)
Anhui	Huangyelianqiao	Zhang et al. (2020)
Yunnan	Fanchuan Cao	Zhang et al. (2020)

*H*. *sampsonii*, which look like gold ingot (Yuanbao in Chinese) by the connate perfoliate leaves, is a perennial herb with a height of approximately 0.2–0.8 m. The stem is erect and glabrous, with slender and short fibrous roots. The upright stem is cylindroid, and the upper part is branched. The shape of leaves is oblong to lanceolate or oblanceolate, (2–) 2.5–7 (8) cm long, (0.7–) 1–3.5 cm wide, and the apex is obtuse or rounded, sessile, with entire margins. Leaves are arranged in opposite and basally completely connate, green above and light green below with dense marginal black glands. The leaf midrib passes through the leaf apex, and both sides have four lateral veins oblique upward; the vein network is fine and sparse. Its inflorescences like corymbose, terminal, which form into the cylindrical panicle; the bracts and bractlets are linear-lanceolate or linear, 4 mm long, with an acuminate apex. The flowers are 6–10 (−15) mm in diameter, nearly oblate, and cup-shaped at the base; the buds are ovate with an obtuse apex. The pedicel is slender and 2–3 mm long; the sepals are oblong to oblong-spatulate or oblong-linear with 3–7 (–10) mm in length and 1–3 mm in width; the petals are light yellow, ovate, persistent, about 4–8 (−13) mm long and 1.5–4 (−7) mm wide, with marginal sessile or nearly sessile black glands. There are three stamens, each containing 10–14 stamens; the anther is light yellow with black glands. The ovary is ovoid to narrowly conical, three-celled, and about 3 mm long; the style is 3, about 2 mm long, separated from the base. The capsule is ovate with a length of 6–9 mm and covered with yellowish-brown glands; seeds are small long ovate, about 1 mm long with yellowish-brown. The fluorescence duration is from May to June and the fruiting period is from July to August. The whole plant is collected in summer or autumn, dried, and used for medicinal purposes (Editorial Committee of Flora of China, 1990; Xie, 2014; Li et al., 2019).

*H*. *sampsonii* not only favors a warm and humid environment but also tolerant to cold and drought. This plant is usually living on hillsides, roadsides, scrub, grassland, fields, and ditches, with an altitude of 0–1,200 m. It is commonly distributed in the south of the Yangtze River and Taiwan in China, and is also found in Japan, Northern Viet Nam, Eastern Myanmar, and Northeast India. Guangxi, Jiangsu, Zhejiang, and Sichuan are major provinces producing this plant (Editorial Committee of Flora of China, 1990).

## 4 Traditional uses

*H*. *sampsonii* has been traditionally used as a folk medicine for the treatment of gastrointestinal diseases and traumatic bleeding in China. The medicinal use of this plant was first recorded in the book of *Ben Cao Cong Xin* (本草从新) in Qing Dynasty, which proposed that it tastes acrid, is cold in nature, functions as nourishing Yin, and can be used to treat haematemesis and epistaxis (Wu, 1957). Textual Research on other monographs of Materia Medica, such as *Bai Cao Jing* and *An Illustrated Book on Plants*, records that the plant can be used for treating carbuncle due to toxins, traumatic injury, and deep-rooted breast carbuncles. It has the functions of clearing heat and detoxifying, relaxing tendons and activating collaterals, cooling blood and stopping bleeding. Clinically, *H*. *sampsonii* has been used to treat a variety of diseases, such as dysentery, enteritis, infantile fever, infantile convulsion, haematemesis, epistaxis, irregular menstruation, leucorrhea, traumatic bleeding, wounds, mastitis, burns, bedsores, and snakebites, etc (Editorial Board of Chinese Materia Medica, 1999; Xie, 2014; Xu, 2016; Vincent et al., 2021). According to the *Gu Shang Zhong Cao Yao Shi Yong Tu Ce* (Li et al., 2019), the fresh herb is often processed by pounding or grinding and used to treat traumatic injuries, gouty arthritis, and finger sores. In addition to external application, the whole plant is generally made into a decoction and taken orally for the treatment of rheumatic arthralgia, hemoptysis due to pulmonary trauma, stranguria due to hematuria, dysmenorrhea, and aphthous ulcers, etc. As a common folk medicine, the medicinal uses of *H*. *sampsonii* are documented in many local medicinal classics (Table 2). For example, *Hunan Yaowu Zhi* recorded that the whole plant of *H*. *sampsonii* can be used to treat diarrhea.

**TABLE 2:** The traditional and clinical uses of *H. sampsonii* in China.

Composition^a^	Dosage form	Traditional and clinical uses	Ref
元宝草(whole plant of *Hypericum sampsonii* Hance)	Unrecorded	Treat haematemesis and epistaxis	*Bencao Congxin*
			《本草从新》
元宝草(whole plant of *Hypericum sampsonii* Hance)	Decoction, and external use	Treat irregular menstruation and relieve itching	*Fenlei Caoyaoxing*
			《分类草药性》
元宝草(whole plant of *Hypericum sampsonii* Hance)	Decoction, and external use	Treat diarrhea, thrush, nebula, burns, cuts, measles without adeqrate eruption, infantile rectocele, and lactation disturbance	*Hunan Yaowu Zhi*
			《湖南药物志》
元宝草(whole plant of *Hypericum sampsonii* Hance), 金银花(flower of *Lonicera japonica* Thunb.), 白头翁(roots of *Pulsatilla chinensis* (Bunge) Regel), 夏枯草(whole plant of *Prunella vulgaris* Linn., 酢浆草(whole plant of *Oxalis corniculata* L.), 油茶(leaves of *Camellia oleifera* Abel)	External use	Treat aphthosis	*Hunan Yaowu Zhi*
			《湖南药物志》
元宝草(whole plant of *Hypericum sampsonii* Hance), 淫羊藿(leaves of *Epimedium brevicornu* Maxim), 油松(*Pinus tabulaeformis* Carr.).	Decoction	Treat low back pain	*Hunan Yaowu Zhi*
			《湖南药物志》
元宝草(whole plant of *Hypericum sampsonii* Hance),车前子(seeds of *Plantago asiatica* L.), *Gardenia jasminoides* Ellis, *Akebia quinata* (Houtt.) Decne	Decoction	Treat leucorrhea	*Hunan Yaowu Zhi*
			《湖南药物志》
忍冬藤(stem of *Lonicera japonica* Thunb), 野菊花(flower of *Dendranthema indicum* (L.) Des Moul.),元宝草(whole plant of *Hypericum sampsonii* Hance), 冰片(borneol)	External use	Treat wounds fester	*Hunan Yaowu Zhi*
			《湖南药物志》
元宝草(whole plant of *Hypericum sampsonii* Hance), 东方狗脊(root of *Woodwardia orientalis* Sw.), 四块瓦(leaves of *Chloranthus serratus* (Thunb.) Roem.), 槟榔(fruits of Areca catechu Linn.)	External use	Treat abdominal pain due to worm	*Hunan Yaowu Zhi*
			《湖南药物志》
元宝草(whole plant of *Hypericum sampsonii* Hance), 蜂蜜(honey)	Decoction	Treat erythral and leukal dysentery, and tenesmus	*Zhejiang Minjian Caoyao*
			《浙江民间草药》
元宝草(whole plant of *Hypericum sampsonii* Hance), 大枣(fruits of *Ziziphus jujuba* Mill.)	Decoction	Treat cough due to Yin dificiency	*Zhejiang Minjian Caoyao*
			《浙江民间草药》
元宝草(whole plant of *Hypericum sampsonii* Hance)	External application	Treat snake bites and finger sores	*Zhejiang Minjian Caoyao*
			《浙江民间草药》
元宝草(whole plant of *Hypericum sampsonii* Hance), 水苏(whole plant of *Stachys japonica* Miq.), 灯笼草(whole plant of *Clinopodium polycephalum* (Vaniot) C. Y. Wu et Hsuan ex Hsu), 筋骨草(whole plant of *Ajuga ciliata* Bunge), 玄参(roots of *Scrophularia ningpoensis* Hemsl.)	Decoction	Treat chronic pharyngitis and hoarseness	*Zhejiang Minjian Changyong Caoyao*
			《浙江民间常用草药》
元宝草(whole plant of *Hypericum sampsonii* Hance), 白酒(alcohol),黄酒(rice wine)	Decoction, and external use	Treat traumatic injury	*Jiangxi Minjian Caoyao*
			《江西民间草药》
元宝草(whole plant of *Hypericum sampsonii* Hance), 白酒(alcohol)	Decoction	Treat breast carbuncle	*Jiangxi Minjian Caoyao*
			《江西民间草药》
元宝草(whole plant of *Hypericum sampsonii* Hance), 商陆(roots of *Phytolacca acinosa* Roxb.), 白酒(alcohol)	Steeping wine	Treat irregular menstruation	*Guizhou Minjian Fangyaoji*
			《贵州民间方药集》
元宝草(whole plant of *Hypericum sampsonii* Hance), 马鞭草(whole plant of *Verbena officinalis* Linn.)	Decoction	Treat lochia	*Guizhou Minjian Fangyaoji*
			《贵州民间方药集》
元宝草(whole plant of *Hypericum sampsonii* Hance), 长春花(flowers of *Catharanthus roseus* (L.) G. Don.), 川芎(roots of *Ligusticum chuanxiong* Hort.)	Steeping wine	Treat menstrual pain	*Guizhou Minjian Fangyaoji*
			《贵州民间方药集》
元宝草(whole plant of *Hypericum sampsonii* Hance), 猪肉(pork)	Decoction	Treat hemoptysis	*Quanzhou Bencao*
			《泉州本草》
长春花(flowers of *Catharanthus roseus* (L.) G. Don.), 益母草(whole plant of *Leonurus japonicus* Houtt.), 元宝草(whole plant of *Hypericum sampsonii* Hance), dry wines	Decoction	Treat irregular menstruation	*Chongqing Caoyao*
			《重庆草药》
大叶仙茅(roots of *Curculigo capitulata* Kuntze), 萱草根(roots of *Hemerocallis fulva* (L.) L.), 女贞子(fruits of *Ligustrum lucidum* W.T.Aiton), 异叶鼠李(roots of *Rhamnus heterophylla* Oliv.), 茺蔚子(seeds of *Leonurus japonicus* Houtt.), 元宝草(whole plant of *Hypericum sampsonii* Hance), 金樱子(seeds of *Rosa laevigata* Michx.), 大枣(fruits of *Ziziphus jujuba* Mill.)	Stewing with chicken	Treat irregular menstruation	*Sichuan Zhongyao Zhi*
			《四川中药志》
四叶葎(whole plant of *Galium bungei* Steud.), 地锦草(whole plant of *Euphorbia humifusa* Willd.), 元宝草(whole plant of *Hypericum sampsonii* Hance), 地耳草(whole plant of *Hypericum japonicum* Thunb.), 马鞭草(whole plant of *Verbena officinalis* Linn.), 酢浆草(whole plant of *Oxalis corniculata* L.), 鹅不食草(*Centipeda minima* (L.) A.Braun & Asch.), 天胡荽(*Hydrocotyle sibthorpioides* Lam.), 飞扬草(whole plant of *Euphorbia hirta* Linn.), 半边莲(whole plant of *Lobelia chinensis* Lour.), 白花蛇舌草(whole plant of *Hedyotis diffusa* Willd.), 墨旱莲(whole plant of *Eclipta prostrata* (L.) L.), 鬼针草(whole plant of *Bidens Pilosa* L.), 野甘草(*Scoparia dulcis* L.), 海金沙(*Lygodium japonicum* (Thunb.) Sw.)	Powder, decoction, or external application	Treat soft tissue contusion, traumatic fracture, postoperative infection, snake bites, burns, appendicitis, nephritis, hepatitis, cholecystitis, pancreatitis, etc	Yu (1995)
柴胡(roots of *Bupleurum chinense* DC.), 白芍(roots of *Paeonia lactiflora* Pall.), 郁金(roots of *Curcuma aromatica* Salisb.), 枳实(fruits of *Citrus aurantium* L.), 白术(roots of *Atractylodes macrocephala* Koidz.), 茯苓(*Poria cocos* (Schw.)Wolf), 陈皮(seedcase of *Citrus reticulata* Blanco), 元宝草(whole plant of *Hypericum sampsonii* Hance)*,* 贯叶连翘(leaves of *Hypericum perforatum* L.), 甘草(roots of *Glycyrrhiza uralensis* Fisch.)	Decoction	Treat generalized anxiety disorder due to Liver-qi stagnation	Chen (2020)
柴胡(roots of *Bupleurum chinense* DC.), 贯叶连翘(leaves of *Hypericum perforatum* L.), 元宝草(whole plant of *Hypericum sampsonii* Hance)*,* 当归(roots of *Angelica sinensis* (Oliv.) Diels), 白芍(roots of *Paeonia lactiflora* Pall.), 川芎(roots of *Ligusticum chuanxiong* Hort.), 茯苓(*Poria cocos* (Schw.)Wolf), 白术(roots of *Atractylodes macrocephala* Koidz.), 酸枣仁(fruits of *Ziziphus jujuba* var. spinosa (Bunge) Hu ex H.F.Chow), 知母(roots of *Anermarrhena asphodeloides* Bunge), 远志(roots of *Polygala tenuifolia* Willd.), 甘草(roots of *Glycyrrhiza uralensis* Fisch.).	Decoction	Treat Generalized Anxiety Disorder	Lin (2018)

^a^ All of the plant names have been checked with “World Flora Online” (www.worldfloraonline.org) mentioning the data of accessing that website.

*H*. *sampsonii* was also widely utilized as ethnomedicine by national minorities in China. In Sandu Shui Autonomous County located in the south of Guizhou Province of China, the botanical drug was commonly used as the liquor fermentation starter by the Shui people. Besides the edible value, this wild plant also possesses a wide range of medicinal values and can be used to treat irregular menstruation, leucorrhea, dysentery, and fever (Hong et al., 2015b). Furthermore, it was often used as an herbal tea to treat gynaecopathia by the Yao minority (Jin et al., 2018). *H*. *sampsonii* was also the medicinal plant traditionally used by Mulam people in Guangxi Province. It was mainly used to treat internal hemorrhage, abnormal menstruation, dysmenorrhea, and bleeding wound (Hu et al., 2020). According to the ethnobotanical data collected from the Maonan minority, *H*. *sampsonii* was used for the treatment of traumatic injury, pain, indigestion, chest congestion, and acute icteric hepatitis (Hong et al., 2015a; Xiang et al., 2018).

Additionally, the whole plant of *H*. *sampsonii* is most frequently reported as a traditional treatment for various diseases. Commonly, two processing methods (internal use and external application) are used before clinical use or self-medication. Firstly, it is processed by removing impurities and non-medicinal parts together with auxiliary materials such as honey and alcohol. Subsequently, the dried or fresh herb is often made as a decoction for oral administration to treat various diseases. Furthermore, in the second step, the dried herb can be ground or freshly pounded, and applied to the affected area for external use.

## 5 Phytochemistry

Chemical investigation of the *Hypericum* species include a series of phloroglucinol derivatives, naphthodianthrones, xanthones, flavonoids, and other phenols and terpenoids (Zhang et al., 2020). Of these, phloroglucinol derivatives are the main secondary metabolites. *H*. *sampsonii* is a rich source of natural products with diverse chemical structures. To date, a total of 223 metabolites including polycyclic polyprenylated acylphloroglucinols (PPAPs), benzophenones, xanthones, flavonoids, bisanthraquinones, and anthraquinones have been separated and identified from *H*. *sampsonii* (Supplementary Figure S10).

### 5.1 Polycyclic polyprenylated acylphloroglucinols (PPAPs)

Phloroglucinols, a type of natural products showing strong oxidizing properties, variable stereochemical structures, and a wide range of pharmacological activities, are decorated with isoprenyl and hydroxyl groups which are substituted at multiple positions on the benzene ring or fused together to form a ring (Xiao and Mu, 2007). PPAPs were highly oxygenated acylphloroglucinol derivatives which decorated with complicated side chains. In the past decades, PPAPs have received extensive attention due to their considerable structural diversity and remarkable biological activities (Yang et al., 2018). Biogenetically, all PPAPs which are derived from a common biosynthetic pathway via different cyclizations of the less complex monocyclic polyprenylated acylphloroglucinols, are generated via three main biosynthetic pathways (Yang et al., 2018). Interestingly, this special class of phloroglucinols has been exclusively isolated from the plants of family Guttiferae (Clusiaceae) and mainly from the genera *Hypericum* and *Garcinia*. Up to December 2022, 116 PPAPs comprise the major family of metabolites identified from *H. sampsonii* (Tables 3, 4, 5). All of the PPAP profiles are generated via three major biosynthetic pathways and may be divided into three groups according to their different scaffolds. Group I are the bicyclic polyprenylated acylphloroglucinols (BPAPs) with major bicyclo [3.3.1]nonane-2,4,9-trione core and related seco-BPAPs. Group II include the caged PPAPs with adamantane (tricyclo [3.3.1.1]decane) and homoadamantane (tricyclo [4.3.1.1]undecane) skeletons. Group III contain other biosynthetically related derivatives which derived from direct cyclizations of monocyclic polyprenylated acylphloroglucinols (MPAPs).

**TABLE 3:** Bicyclic Polyprenylated Acylphloroglucinols (BPAPs) isolated from *H. sampsonii*.

No.	Compound name	Molecular formula	Molecular weight	Part of the plant	Ref
1	7-Epiclusianone	C_33_H_42_O_4_	502.70	Root	Xiao et al. (2007)
2	Attenuatumione C	C_38_H_50_O_5_	586.81	Aerial part	Zhu et al. (2015)
3	Hyperattenin C	C_38_H_50_O_5_	586.81	Aerial part	Zhang et al. (2016)
4	Hyperattenin E	C_33_H_43_O_5_	519.70	Aerial part	(Tian, 2015)]
5	Hyperforatin F	C_33_H_48_O_5_	524.74	Whole plant	Chen et al. (2020)
6	Hyperforin	C_35_H_52_O_4_	536.80	Aerial part	Zheng et al. (2003)
7	Hyperibone A	C_33_H_42_O_5_	518.69	Aerial part	Zhang et al. (2016)
8	Hyperibone I	C_33_H_42_O_5_	518.69	Aerial part	Tian (2015)
9	Hypersampsone F	C_38_H_48_O_4_	568.80	Aerial part	Lin and Wu (2003)
10	Hypersampsone H	C_38_H_50_O_4_	570.80	Fruit	Zeng et al. (2009)
11	Hypersampsone K	C_38_H_50_O_4_	570.80	Fruit	Zeng et al. (2012)
12	Otogirinin D	C_38_H_50_O_5_	586.81	Aerial part	Zhu et al. (2015)
13	Otogirinin E	C_38_H_50_O_6_	602.81	Aerial part	Tian (2015)
14	Hyperisampsin H	C_35_H_42_O_6_	558.72	Aerial part	Zhu et al. (2017b)
15	Hyperisampsin I	C_35_H_42_O_6_	558.72	Aerial part	Zhu et al. (2017b)
16	Hyperisampsin J	C_38_H_50_O_7_	618.81	Aerial part	Zhu et al. (2017b)
17	Hyperisampsin K	C_38_H_50_O_8_	634.81	Aerial part	Zhu et al. (2017b)
18	Hyperisampsin L	C_38_H_50_O_8_	634.81	Aerial part	Zhu et al. (2017b)
19	Hyperisampsin M	C_38_H_50_O_7_	618.81	Aerial part	Zhu et al. (2017b)
20	Hypersampsone R^T^	C_30_H_36_O_4_	460.61	Aerial part	Tian et al. (2014b)
21	Hypersampsone R^C^	C_32_H_42_O_3_	474.68	Aerial part	Chen et al. (2014)
22	Hypersampsone S^T^	C_38_H_50_O_5_	586.81	Aerial part	Tian et al. (2016)
23	Hypersampsone T	C_33_H_42_O_4_	502.70	Aerial part	Tian et al. (2016)
24	Hypersampsone U	C_33_H_42_O_5_	518.69	Aerial part	Tian et al. (2016)
25	Hypersampsone V	C_33_H_44_O_7_	552.71	Aerial part	Tian et al. (2016)
26	Hypersampsone W	C_33_H_44_O_7_	552.71	Aerial part	Tian et al. (2016)
27	Sampsonione K	C_38_H_50_O_5_	586.81	Aerial part	Hu and Sim (2000)
28	Sampsonione L	C_33_H_42_O_5_	518.69	Aerial part	Hu and Sim (2000)
29	Sampsonione M	C_38_H_50_O_5_	586.81	Aerial part	Hu and Sim (2000)
30	Sampsonione N	C_33_H_42_O_5_	518.69	Root	Xiao et al. (2007)
31	Sampsonione O	C_33_H_42_O_5_	518.69	Root	Xiao et al. (2007)
32	Sampsonione P	C_33_H_42_O_5_	518.69	Root	Xiao et al. (2007), Huang et al. (2022)

^T^ and ^C^ These compound names are distinguished by the initials of authors because of their different structures while identical names.

**TABLE 4:** Caged PPAPs isolated from *H. sampsonii*.

No.	Compound name	Molecular formula	Molecular weight	Part of the plant	Ref
33	28,29-Epoxyplukenetione A	C_33_H_40_O_5_	516.68	Aerial part	Zhu et al. (2014)
34	Attenuatumione D	C_38_H_50_O_5_	586.81	Aerial part	Zhang et al. (2016)
35	Cowabenzophenone B	C_35_H_44_O_5_	544.73	Aerial part	Zhang et al. (2016)
36	Dioxasampsone A	C_33_H_42_O_6_	534.69	Aerial part	Tian et al. (2014b)
37	Dioxasampsone B	C_33_H_42_O_7_	550.69	Aerial part	Tian et al. (2014b)
38	Hyperattenin G	C_35_H_42_O_5_	542.72	Aerial part	Zhang et al. (2016)
39	Hyperattenin I	C_38_H_50_O_6_	602.81	Aerial part	Zhang et al. (2016)
40	Hypercohone A	C_33_H_42_O_5_	518.69	Aerial part	Chen et al. (2014)
41	Hyperibone K	C_33_H_40_O_4_	500.70	Aerial part	Chen et al. (2014)
42	Hypericumone A	C_32_H_40_O_4_	488.67	Aerial part	Huang et al. (2020)
43	Hyperisampsin A	C_38_H_50_O_6_	602.81	Aerial part	Zhu et al. (2015)
44	Hyperisampsin B	C_38_H_50_O_5_	586.81	Aerial part	Zhu et al. (2015)
45	Hyperisampsin C	C_38_H_48_O_5_	584.80	Aerial part	Zhu et al. (2015)
46	Hyperisampsin D	C_38_H_50_O_7_	618.81	Aerial part	Zhu et al. (2015)
47	Hyperisampsin E	C_33_H_40_O_5_	516.68	Aerial part	Zhu et al. (2015)
48	Hyperisampsin F	C_33_H_42_O_6_	534.69	Aerial part	Zhu et al. (2015)
49	Hyperisampsin G	C_38_H_48_O_5_	584.80	Aerial part	Zhu et al. (2015)
50	Hyperisampsin N	C_38_H_50_O_7_	618.81	Aerial part	Zhu et al. (2017b)
51	Hyperisampsin O	C_38_H_50_O_8_	634.81	Aerial part	Zhu et al. (2017b)
52	Hypersampsone A	C_35_H_50_O_4_	534.78	Aerial part	Lin and Wu (2003)
53	Hypersampsone B	C_35_H_52_O_4_	536.80	Aerial part	Lin and Wu (2003)
54	Hypersampsone C	C_32_H_46_O_4_	494.72	Aerial part	Lin and Wu (2003)
55	Hypersampsone D	C_38_H_50_O_4_	570.80	Aerial part	Lin and Wu (2003)
56	Hypersampsone E	C_38_H_50_O_4_	570.80	Aerial part	Lin and Wu (2003)
57	Hypersampsone G	C_38_H_50_O_4_	570.80	Fruit	Zeng et al. (2009)
58	Hypersampsone I	C_35_H_44_O_4_	528.73	Fruit	Zeng et al. (2012a)
59	Hypersampsone J	C_38_H_48_O_4_	568.80	Fruit	Zeng et al. (2012a)
60	Hypersampsone L	C_38_H_50_O_4_	570.80	Fruit	Zeng et al. (2012a)
61	Hypersampsone M	C_30_H_36_O_4_	460.61	Aerial part	Tian et al. (2014c)
62	Hypersampsone N	C_30_H_36_O_6_	492.61	Aerial part	Tian et al. (2014a)
63	Hypersampsone O	C_33_H_40_O_5_	516.68	Aerial part	Tian et al. (2014a)
64	Hypersampsone P	C_30_H_36_O_4_	460.61	Aerial part	Tian et al. (2014a)
65	Hypersampsone Q	C_33_H_42_O_5_	518.69	Aerial part	Tian et al. (2014a)
66	Hypersampsone S^C^	C_32_H_46_O_4_	494.72	Aerial part	Chen et al. (2014)
67	Hypersampsone X	C_33_H_40_O_4_	500.70	Aerial part	Tian et al. (2017b)
68	Hypersampsonone A	C_38_H_50_O_5_	586.81	Aerial part	Zhang et al. (2016)
69	Hypersampsonone B	C_35_H_44_O_6_	560.73	Aerial part	Zhang et al. (2016)
70	Hypersampsonone C	C_38_H_50_O_7_	618.81	Aerial part	Zhang et al. (2016)
71	Hypersampsonone D	C_39_H_52_O_6_	616.84	Aerial part	Zhang et al. (2016)
72	Hypersampsonone E	C_35_H_44_O_6_	560.73	Aerial part	Zhang et al. (2016)
73	Hypersampsonone F	C_38_H_50_O_6_	602.81	Aerial part	Zhang et al. (2016)
74	Hypersampsonone G	C_38_H_50_O_5_	586.81	Aerial part	Zhang et al. (2016)
75	Hyphenrone N	C_38_H_50_O_6_	602.81	Aerial part	Zhang et al. (2016)
76	Norsampsone E	C_29_H_42_O_4_	454.65	Aerial part	Tian et al. (2017b)
77	Otogirinin A	C_38_H_49_O_4_	569.81	Aerial part	Huang et al. (2020)
78	Otogirinin B	C_38_H_50_O_7_	618.81	Aerial part	Zhu et al. (2017b)
79	Otogirinin C	C_38_H_50_O_5_	586.81	Aerial part	Zhu et al. (2017b)
80	Peroxysampsone A	C_33_H_42_O_8_	566.69	Root	Xiao et al. (2010)
81	Peroxysampsone B	C_33_H_42_O_7_	550.69	Root	Xiao et al. (2010)
82	Plukenetione B	C_33_H_42_O_5_	518.69	Aerial part	Chen et al. (2014)
83	Plukenetione A	C_33_H_40_O_4_	500.70	Aerial part	Tian (2015)
84	Plukenetione C	C_33_H_42_O_7_	550.69	Root	Xiao et al. (2010)
85	Sampsonione A	C_38_H_50_O_5_	586.81	Aerial part	Hu and Sim (1998)
86	Sampsonione B	C_33_H_42_O_5_	518.69	Aerial part	Hu and Sim (1998)
87	Sampsonione C	C_38_H_50_O_5_	586.81	Aerial part	Hu and Sim (1999b)
88	Sampsonione D	C_38_H_48_O_4_	568.80	Aerial part	Hu and Sim (1999b)
89	Sampsonione E	C_35_H_42_O_5_	542.72	Aerial part	Hu and Sim (1999b)
90	Sampsonione F	C_38_H_50_O_5_	586.81	Aerial part	Hu and Sim (1999b)
91	Sampsonione G	C_33_H_42_O_5_	518.69	Aerial part	Hu and Sim (1999b)
92	Sampsonione H	C_35_H_44_O_4_	528.73	Aerial part	Hu and Sim (1999b)
93	Sampsonione I	C_38_H_48_O_5_	584.80	Aerial part	Hu and Sim (1999a)
94	Sampsonione J	C_38_H_48_O_5_	584.80	Aerial part	Hu and Sim (1999a)
95	Sampsonione Q	C_33_H_40_O_5_	516.68	Root	Xiao et al. (2007)
96	Sampsonione R	C_30_H_36_O_5_	476.61	Root	Xiao et al. (2007)

**TABLE 5:** Other PPAPs isolated from *H. sampsonii*.

No.	Compound name	Molecular formula	Molecular weight	Part of the plant	Ref
97	Hyperhexanone A	C_39_H_54_O_6_	618.86	Aerial part	Zhu et al. (2016b)
98	Hyperhexanone B	C_30_H_42_O_2_	434.66	Aerial part	Zhu et al. (2016b)
99	Hyperhexanone F	C_34_H_46_O_6_	550.74	Aerial part	Zhang et al. (2021)
100	Hypsampsone A	C_33_H_40_O_6_	532.68	Aerial part	Zhang et al. (2021)
101	Norhypersampsone A	C_20_H_24_O_3_	312.41	Aerial part	Zhang et al. (2017)
102	Norsampsone A	C_32_H_44_O_3_	476.70	Aerial part	Tian et al. (2014c)
103	Norsampsone B	C_32_H_44_O_3_	476.70	Aerial part	Tian et al. (2014c)
104	Norsampsone C	C_37_H_52_O_3_	544.82	Aerial part	Tian et al. (2014c)
105	Norsampsone D	C_37_H_52_O_3_	544.82	Aerial part	Tian et al. (2014c)
106	Hypericumone B	C_30_H_42_O_2_	434.66	Aerial part	Huang et al. (2020)
107	Sampsone A	C_22_H_24_O_6_	384.43	Aerial part	Xin et al. (2011b)
108	Sampsonol A	C_33_H_42_O_7_	550.69	Aerial part	Xin et al. (2012)
109	Sampsonol B	C_33_H_42_O_7_	550.69	Aerial part	Xin et al. (2012)
110	Sampsonol C	C_29_H_34_O_5_	462.59	Aerial part	Xin et al. (2012)
111	Sampsonol D	C_29_H_34_O_6_	478.59	Aerial part	Xin et al. (2012)
112	Sampsonol E	C_27_H_38_O_5_	442.60	Aerial part	Xin et al. (2012)
113	Sampsonol F	C_26_H_36_O_5_	428.57	Aerial part	Xin et al. (2012)
114	Hypersampson A	C_37_H_50_O_4_	558.80	Aerial part	Huang et al. (2022)
115	Hypersampson B	C_37_H_50_O_4_	558.80	Aerial part	Huang et al. (2022)
116	Hypersampson C	C_37_H_50_O_4_	558.80	Aerial part	Huang et al. (2022)

#### 5.1.1 Bicyclic polyprenylated acylphloroglucinols (BPAPs)

The bicyclic polyprenylated acylphloroglucinols (BPAPs) with major bicyclo [3.3.1] nonane-2,4,9-trione core and related seco-BPAPs (**1**–**32**, Table 3) include 32 BPAPs in which the acyl group is located at the C-1 or C-3 position. In 2000, BPAPs (Sampsoniones K-M, **27**-**29**) were first discovered in the ethanolic extract of the aerial parts of *H*. *sampsonii* (Hu and Sim, 2000). Since then, BPAPs have been increasingly explored from the roots, fruits, and aerial part of *H. sampsonii*.

#### 5.1.2 Caged PPAPs with adamantane or homoadamantane skeletons

It is noteworthy that *H. sampsonii* is a rich source of caged PPAPs, and about 64 adamantane- and homoadamantane-type derivatives with adamantane (tricyclo [3.3.1.1]decane) and homoadamantane (tricyclo [4.3.1.1]undecane) (**33**–**96**, Table 4) have also been isolated from this plant. As early as 1998, Hu and Sim isolated two caged PPAPs (sampsoniones A and B, **85–86**) from the aerial parts of *H. sampsonii* (Hu and Sim, 1998). Subsequently, they discovered sampsoniones C-J (**87–94**) (Hu and Sim, 1999a; b, 2000). A few years later, sampsoniones Q-R (**95–96**) were isolated from the root of *H. sampsonii* (Xiao et al., 2007). Since then, a large number of studies of caged PPAPs which were isolated from *H. sampsonii* have been reported, primarily focusing on its structure diversity with an unprecedented carbon skeleton. The tetracyclo [6.3.1.1(3,10).0(3,7)]tridecane skeletons and biogenetically related congeners, such as 28,29-Epoxyplukenetione A (**33**) (Zhu et al., 2014), hyperisampsins A-G (**43–49**) (Zhu et al., 2014), hyperisampsins N (**50**), and hyperisampsins O (**51**) (Zhu et al., 2017a), hypersampsones A-E (**52–56**) (Lin and Wu, 2003), hypersampsones L-S (**60–66**) (Zeng et al., 2012b), and hypersampsonones A-G (**68–74**) (Zhang et al., 2016).

#### 5.1.3 Other PPAPs

A total of 20 other PPAPs such as spirocyclic PPAPs with octahydrospiro-[cyclohexan-1,5′-indene] core and complicated PPAPs via intramolecular [4 + 2] cycloadditions from MPAPs (**97**-**116**, Table 5) have been isolated from *H. sampsonii*. In 2011, a novel prenylated aromatic lactone (sampsone A, **107**) was isolated from the aerial parts of *H. sampsonii* (Xin et al., 2011). Soon afterwards, six new acylphloroglucinol derivatives, (sampsonols A-F, **108–113**), were discovered from the aerial parts of *H. sampsonii* (Xin et al., 2012). In 2014, four new decarbonyl PPAPs, (norsampsones A-D, **102**-**105**), were isolated from the 60% EtOH extract of the aerial parts of *H. sampsonii* (Tian et al., 2014c). Recently, three nor-polycyclic polyprenylated acylphloroglucinols with a tetracyclic 6/5/5/6 ring system, (Hypersampones A-C, **114**-**116**), which showed a lipid-lowering activity, were isolated from *H. sampsonii*. (Huang et al., 2022).

### 5.2 Benzophenones

Natural benzophenone derivatives have attracted extensive attention due to their unique structures and extensive biological activities. In accordance with the literature, benzophenones, mainly including simple benzophenone derivatives (SBDS) and polyprenylated benzophenones (PPBS), may be the precursors of some xanthones (Kitanov and Nedialkov, 2001). Currently, there are 33 benzophenones isolated from *H. sampsonii* (Supplementary Figure S4; Table 6). Among them, two pairs of racemic PPBS, (±)-sampsonin A-B (**117–120**) were chirally separated from *H. sampsonii* (Tian et al., 2017a). In addition, seven benzophenone derivatives sampbenzophenones A-G (**140–146**) were isolated from the aerial parts of *H. sampsonii* (Zhu et al., 2016a).

**TABLE 6:** Benzophenones isolated from *H. sampsonii*.

No.	Compound name	Molecular formula	Molecular weight	Part of the plant	Ref
117	(−)-Sampsonin A	C_28_H_32_O_4_	432.56	Aerial part	Tian et al. (2017a)
118	(−)-Sampsonin B	C_28_H_32_O_4_	432.56	Aerial part	Tian et al. (2017a)
119	(+)-Sampsonin A	C_28_H_32_O_4_	432.56	Aerial part	Tian et al. (2017a)
120	(+)-Sampsonin B	C_28_H_32_O_4_	432.56	Aerial part	Tian et al. (2017a)
121	(E)-3-(3,7-dimethylocta-2,6-dienyl)-2,4,6-trihydroxybenzophenone	C_23_H_26_O_4_	366.46	Aerial part	Zhang et al. (2017)
122	(Z)-3-(3,7-dimethylocta-2,6-dienyl)-2,4,6-trihydroxybenzophenone	C_23_H_26_O_4_	366.46	Aerial part	Zhang et al. (2017)
123	2,4,6,3′,5′-Pentamethoxylbenzophenone	C_18_H_20_O_6_	332.35	Aerial part	Qiu et al. (2015)
124	2,4,6-Trihydroxybenzophenone	C_13_H_10_O_4_	230.22	Aerial part	Lin and Wu (2003)
125	2,4,6-Trihydroxybenzophenone 3-C-geranyl ether	C_23_H_26_O_4_	366.46	Aerial part	Lin and Wu (2003)
126	2,4,6-Trihydroxybenzophenone 4-O-geranyl ether	C_23_H_26_O_4_	366.46	Aerial part	Lin and Wu (2003)
127	2,6-Dihydroxy-4,3′,5′-trihymethoxy-benzophenone	C_16_H_16_O_6_	304.30	Whole plant	Yin et al. (2013)
128	2,6-Dihydroxy-4-[(E)-5-hydroxy-3,7-dimethylocta-2,7-dienyloxy]-benzophenone	C_23_H_26_O_5_	382.46	Whole plant	Don et al. (2004)
129	2,6-Dihydroxy-4-[(E)-7-hydroxy-3,7-dimethylocta-2-enyloxy]-benzophenone	C_23_H_28_O_5_	384.46	Whole plant	Don et al. (2004)
130	2-Hydroxy-4,6-dimethoxybenzophenone	C_15_H_14_O_4_	258.27	Aerial part	Qiu et al. (2015)
131	2-β-D-glucopyranosyl-4,6-dihydroxyphenyl phenyl ketone	C_19_H_20_O_9_	392.36	Aerial part	Hong et al. (2004)
132	3-(2-Hydroxy-7-methyl-3-methyleneoct-6-enyl)-5-isoprenyl-2,4,6-trihydroxybenzophenone	C_28_H_34_O_5_	450.58	Aerial part	Zhang et al. (2017)
133	4-Geranyloxy-2,6-dihydroxybenzophenone	C_23_H_26_O_4_	366.46	Aerial part	Zhu et al. (2016a)
134	4-Geranyloxy-2-hydroxy-6-isoprenyloxybenzophenone	C_28_H_34_O_4_	434.58	Aerial part	Huang et al. (2020)
135	8-Benzoyl-2,2-dimethyl-6-(E-3,7-dimethyl-2,6-octadi-enyl)-3,5,7-trihydroxy chromane	C_28_H_34_O_5_	450.58	Aerial part	Zhu et al. (2016a)
136	Garcimangosone D	C_19_H_20_O_9_	392.36	Whole plant	Chen et al. (2020)
137	Otogirinin F	C_28_H_34_O_5_	450.58	Aerial part	Tian (2015)
138	Otogirinin G	C_28_H_34_O_5_	450.58	Aerial part	Zhang et al. (2017)
139	Petiolin F	C_19_H_20_O_10_	408.36	Whole plant	Dung Nguyen et al. (2021)
140	Sampbenzophenone A^x^	C_28_H_34_O_5_	450.58	Aerial part	Zhu et al. (2016a)
141	Sampbenzophenone B	C_28_H_34_O_5_	450.58	Aerial part	Zhu et al. (2016a)
142	Sampbenzophenone C	C_28_H_34_O_5_	450.58	Aerial part	Zhu et al. (2016a)
143	Sampbenzophenone D	C_23_H_26_O_5_	382.46	Aerial part	Zhu et al. (2016a)
144	Sampbenzophenone E	C_23_H_26_O_6_	398.46	Aerial part	Zhu et al. (2016a)
145	Sampbenzophenone F	C_22_H_26_O_6_	386.44	Aerial part	Zhu et al. (2016a)
146	Sampbenzophenone G	C_23_H_26_O_5_	382.46	Aerial part	Zhu et al. (2016a)
147	Sampsine A	C_16_H_16_O_6_	304.30	Aerial part	Qiu et al. (2015)
148	Sampsine B	C_22_H_26_O_10_	450.44	Aerial part	Qiu et al. (2015)
149	Sampsone F^x^	C_28_H_34_O_5_	450.58	Aerial part	Tian (2015)
150	Sampsone G	C_28_H_34_O_5_	450.58	Aerial part	Tian (2015)

^x^—Two compounds have the same structure while different names.

### 5.3 Xanthones

Xanthones, a class of iso-tricyclic compounds mainly divided into simple xanthones, glycosylated xanthones, prenylated xanthones, and sulfonated xanthones, are known to possess a variety of biological activities, such as antihypertensive, antiviral, and antitumor activities. In addition, the discrepancy in xanthones activity depends on the substituents on the aromatic rings.

In 1985, Chen MT and Chen CM isolated hyperxanthone (**178**) from the whole plant of *H. sampsonii*, and firstly discovered 2-hydroxy-3.4-dimethoxyxanthone (**164**) and isomangiferin (**179**) in the genus Hypericum (Chen and Chen, 1985). Further study on the constituents in the whole plant of *H. sampsonii*, Hong et al. also isolated two xanthone sulfonates, 1,3-dihydroxy-5-methoxyxanthone-4-sulfonate (**158**) and 1,3-dihydroxy-5-O-β-D-glucopyranosylxanthone-4-sulfonate (**159**) (Hong et al., 2004). The reported metabolites and structures of xanthones are shown in Supplementary Figure S5; Table 7.

**TABLE 7:** Xanthones isolated from *H. sampsonii*.

No.	Compound name	Molecular formula	Molecular weight	Part of the plant	Ref
151	1,3,5,6-Tetrahydroxy-2-prenylxanthone	C_18_H_16_O_6_	328.32	Whole plant	Don et al. (2004)
152	1,3,5,6-Tetrahydroxyxanthone	C_13_H_8_O_6_	260.20	Whole plant	Don et al. (2004)
153	1,3,5-Trihydroxy-xanthone	C_13_H_8_O_5_	244.20	Aerial part	Guo et al. (2007)
154	1,3,6,7-Tetrahydroxy-8-(3-methyl-but-2-enyl)-xanthone	C_18_H_16_O_6_	328.32	Whole plant	Li et al. (2004)
155	1,3,6,7-Tetrahydroxy-xanthone (Norathyriol)	C_13_H_8_O_6_	260.20	Whole plant	Don et al. (2004)
156	1,3-Dihydroxy-2-methoxyxanthone	C_14_H_10_O_5_	258.23	Whole plant	Shi et al. (2016)
157	1,3-Dihydroxy-5-methoxyxanthone	C_14_H_10_O_5_	258.23	Whole plant	Dong et al. (2015)
158	1,3-Dihydroxy-5-methoxyxanthone-4-sulfonate	C_14_H_9_O_8_KS	376.38	Whole plant	Hong et al. (2004)
159	1,3-Dihydroxy-5-O-β-D-glucopyranosylxanthone-4-sulfonate	C_19_H_17_O_13_SK	524.49	Whole plant	Hong et al. (2004)
160	1,6-Dihydroxyxanthone	C_13_H_8_O_4_	228.20	Whole plant	Don et al. (2004)
161	1,7-Dihydroxy-2-methoxyxanthone	C_14_H_10_O_5_	258.23	Whole plant	Shi et al. (2016)
162	1,7-Dihydroxy-4-methoxyxanthone	C_14_H_10_O_5_	258.23	Whole plant	Li et al. (2004)
163	1-Hydroxy-7-methoxy-9H-xanthen-9-one	C_14_H_10_O_4_	242.23	Whole plant	Chen et al. (2020)
164	2-Hydroxy-3,4-dimethoxyxanthone	C_15_H_12_O_5_	272.26	Whole plant	Chen and Chen (1985)
165	2-Hydroxy-5-methoxyxanthone	C_14_H_10_O_4_	242.23	Whole plant	Shi et al. (2016)
166	2-Hydroxyxanthone	C_13_H_8_O_3_	212.20	Root	Xiao et al. (2008)
167	2-Methoxy-1,5-dihydroxyxanthone	C_14_H_10_O_5_	258.23	Aerial part	Xin et al. (2011a)
168	2-Methoxyxanthone	C_14_H_10_O_3_	226.23	Aerial part	Zhang et al. (2017)
169	5′-Demethoxycadensin G	C_23_H_18_O_9_	438.39	Whole plant	Chen et al. (2020)
170	5-Methoxy-1,3,7-trihydroxy xanthone	C_14_H_10_O_6_	274.23	Aerial part	Xin et al. (2011a)
171	5-O-methyl-2-deprenyIrheediaxanthone B	C_19_H_18_O_6_	342.35	Whole plant	Chen et al. (2020)
172	7-Methoxy-1,5,6-trihydroxyxanthone	C_15_H_12_O_6_	288.26	Aerial part	Xin et al. (2011a)
173	Euxanthone	C_13_H_8_O_4_	228.20	Aerial part	Hong et al. (2004)
174	Hypericumxanthone A	C_19_H_18_O_6_	342.35	Aerial part	Xin et al. (2011a)
175	Hypericumxanthone B	C_23_H_22_O_6_	394.42	Aerial part	Xin et al. (2011a)
176	Hyperixanthone A	C_28_H_32_O_6_	464.56	Root	Xiao et al. (2008)
177	1,3,5,8-Tetrahydroxy-6-methoxy-7-isoprenylxanthone	C_19_H_18_O_7_	358.35	Whole plant	Don et al. (2004)
178	Hyperxanthone (5,9-Dihydroxy-3,3-dimethylpyrano [3,2-a]xanthen-12-one)	C_18_H_14_O_5_	310.31	Whole plant	Chen and Chen (1985)
179	Isomangiferin	C_19_H_18_O_11_	422.34	Whole plant	Chen and Chen (1985)
180	Jacareubin	C_18_H_14_O_6_	326.30	Whole plant	Chen et al. (2020)
181	Mangiferin	C_19_H_18_O_11_	422.34	Whole plant	Chen and Chen (1985)
182	Neolancerin	C_19_H_18_O_10_	406.34	Whole plant	Don et al. (2004)
183	Padiaxanthone	C_23_H_20_O_6_	392.41	Whole plant	Don et al. (2004)
184	Patulone	C_23_H_24_O_6_	396.44	Whole plant	Li et al. (2004)
185	Sampsone C	C_18_H_18_O_8_	362.33	Aerial part	Xin et al. (2011b)
186	Toxyloxanthone B	C_18_H_14_O_6_	326.30	Whole plant	Chen and Chen (1985)

### 5.4 Flavonoids

Flavonoids, including flavanols, biflavonoids, and common flavonoids, constitute an important class of metabolites in *H*. *sampsonii*. To date, twelve flavonoids have been isolated and identified from *H*. *sampsonii* (Supplementary Figure S6; Table 8). According to the documents, we have found that structures of these metabolites are generally based on the structure quercetin (**193**), in which the groups usually substitute at the 3- and 3′- positions, while all of saccharide groups located at C-3 in flavonoid glycosides.

**TABLE 8:** Flavonoids isolated from *H. sampsonii*.

No.	Compound name	Molecular formula	Molecular weight	Part of the plant	Ref
187	(+)-Catechin	C_15_H_14_O_6_	290.27	Whole plant	Chen et al. (2020)
188	3,8″-Biapigenin	C_30_H_18_O_10_	538.46	Whole plant	Dong et al. (2015)
189	Kaempferol	C_15_H_10_O_6_	286.24	Whole plant	Don et al. (2004)
190	Kaempferol-3-O-glucopyranoside	C_21_H_20_O_11_	448.38	Whole plant	Don et al. (2004)
191	Luteolin	C_15_H_10_O_6_	286.24	Aerial part	Hong et al. (2004)
192	Naringenin	C_15_H_12_O_5_	272.26	Whole plant	Chen et al. (2020)
193	Quercetin	C_15_H_10_O_7_	302.24	Whole plant	Don et al. (2004)
194	Quercetin 3-galactoside (Hyperin, Hyperoside)	C_21_H_20_O_12_	464.38	Whole plant	Zeng et al. (2002)
195	Quercetin 3-O-glucopyranoside	C_21_H_20_O_12_	464.38	Whole plant	Don et al. (2004)
196	Quercetin-3-O-arabinoside	C_20_H_18_O_11_	434.35	Whole plant	Chen et al. (2020)
197	Quercitrin	C_21_H_20_O_11_	448.38	Whole plant	Chen et al. (2020)
198	Rutin	C_27_H_30_O_16_	610.52	Whole plant	Chen et al. (2020)

### 5.5 Naphthodianthrones

Naphthodianthrones, one out of the most biologically active substances in *H. sampsonii*, are mainly represented by hypericin and pseudohypericin (Supplementary Figure S7; Table 9). Hypericin (**199**), the active metabolite isolated from the flowers and fruits of *H. sampsonii*, is considered the characteristic constituent for the identification of this plant (Zeng et al., 2002). Subsequently, pseudohypericin (**200**) was isolated from the aerial parts of *H. sampsonii* (Zheng, 2005).

**TABLE 9:** Naphthodianthrones isolated from *H. sampsonii*.

No.	Compound name	Molecular formula	Molecular weight	Part of the plant	Ref
199	Hypericin	C_30_H_16_O_8_	504.45	Flower and fruit	Zeng et al. (2002)
200	Pseudohypericin	C_30_H_16_O_9_	520.45	Aerial part	Zheng (2005)

### 5.6 Anthraquinones

Anthraquinones found in *H. sampsonii* generally include two types of single anthraquinones and bisanthraquinones. As shown in Supplementary Figure S8, compounds **201–204** are single anthraquinones, while compounds **205–207** are bisanthraquinones. The chemical structures of anthraquinones are listed in Table 10.

**TABLE 10:** Anthraquinones isolated from *H. sampsonii*.

No.	Compound name	Molecular formula	Molecular weight	Part of the plant	Ref
201	1,3,6-Trihydroxy-2-methylanthra-quinone	C_15_H_10_O_5_	270.24	Whole plant	Yin et al. (2013)
202	3-Ethyl-1,8-dihydroxy-6-methoxyanthracene-9,10-dione	C_17_H_14_O_5_	298.29	Whole plant	Chen et al. (2020)
203	Emodin	C_15_H_10_O_5_	270.24	Whole plant	Don et al. (2004)
204	Physcion	C_16_H_12_O_5_	284.27	Whole plant	Qi et al. (2008)
205	R-(−)-skyrin-6-O-β-D-glucopyranoside	C_36_H_28_O_15_	700.61	Whole plant	Don et al. (2004)
206	R-(−)-skyrin-6-O-β-D-xylopyranoside	C_35_H_26_O_14_	670.58	Whole plant	Don et al. (2004)
207	S-(+)-skyrin-6-O-β-D-glucopyranoside	C_36_H_28_O_15_	700.59	Whole plant	Don et al. (2004)

### 5.7 Simple aromatic compounds

Simple aromatic compounds in the extracts of *H. sampsonii* refer to the compounds with a benzene ring, which have simple structure and small relative molecular weight. The main compounds are presented in Supplementary Figure S9; Table 11. Xin WB and his co-works found a rare chemical structure sampsone B (**218**) in the aerial parts of *H. sampsonii* (Xin et al., 2011).

**TABLE 11:** Simple aromatic compounds isolated from *H. sampsonii*.

No.	Compound name	Molecular formula	Molecular weight	Part of the plant	Ref
208	3,4-Dihydroxybenzoic acid	C_7_H_6_O_4_	154.12	Whole plant	Kang et al. (2011)
209	3,4-Dihydroxybenzoic acid ethyl ester	C_9_H_10_O_4_	182.18	Whole plant	Yin et al. (2013)
210	3,4-Dihydroxycinnamic acid	C_9_H_8_O_4_	180.16	Aerial part	Hong et al. (2004)
211	5,7-Dihydroxy-3-methylchromone	C_10_H_8_O_4_	192.17	Whole plant	Chen et al. (2020)
212	Benzoic acid	C_7_H_6_O_2_	122.12	Aerial part	Guo et al. (2005)
213	Caffeic acid methyl ester	C_10_H_10_O_4_	194.19	Whole plant	Shi et al. (2016)
214	Ferulic acid	C_10_H_10_O_4_	194.19	Whole plant	Shi et al. (2016)
215	Gallic acid	C_7_H_6_O_5_	170.12	Whole plant	Chen et al. (2020)
216	Octadecyl ferulate	C_28_H_46_O_4_	446.67	Whole plant	Chen et al. (2020)
217	P-hydroxybenzoic acid	C_7_H_6_O_3_	138.12	Whole plant	Kang et al. (2011)
218	Sampsone B	C_15_H_16_O_6_	292.29	Aerial part	Xin et al. (2011b)
219	Vanillic acid	C_8_H_8_O_4_	168.15	Whole plant	Shi et al. (2016)

### 5.8 Other secondary metabolites

In addition to the aforementioned compounds, other compounds including alkaloids, porphyrins, steroids, pentacyclic triterpenoids and so on have been found in *H. sampsonii*. Chen Q isolated 6-ethoxy-1H-pyrimidine-2,4-dione (**220**) from the whole plant of *H. sampsonii* (Chen et al., 2020). Qi JB and his colleagues found chlorophyll A (**221**) from the extract of *H. sampsonii* (Qi et al., 2008). Additionally, Chen Q also discovered β-sitosterol (**222**) from this plant (Chen et al., 2020). And Guo *et al.* isolated stigmasteol (**223**) from the aerial parts of *H. sampsonii* (Guo et al., 2005). Betulinic acid (**224**), a pentacyclic triterpenoid compound, was also discovered in this botanical drug (Don et al., 2004). Furthermore, this plant was also demonstrated to contain 2-caffeoyloxy-3-hydroxy-3-(3,4-dihydroxyphenyl) propyl alcohol (**225**) (Don et al., 2004), octacosanol (**226**) (Guo et al., 2005), and triacontanoic acid (**227**) (Guo et al., 2005). The variety and structure of other compounds are displayed in Supplementary Figure S10; Table 12.

**TABLE 12:** Other compounds isolated from *H. sampsonii*.

No.	Compound name	Molecular formula	Molecular weight	Part of the plant	Ref
220	6-Ethoxy-1H-pyrimidine-2,4-dione	C_6_H_8_N_2_O_3_	156.14	Whole plant	Chen et al. (2020)
221	Chlorophyll A	C_55_H_72_MgN_4_O_5_	893.51	Whole plant	Qi et al. (2008)
222	β-Sitosterol	C_29_H_50_O	414.72	Whole plant	Chen et al. (2020)
223	Stigmasterol	C_29_H_48_O	412.70	Aerial part	Guo et al. (2005)
224	Betulinic acid	C_30_H_48_O_3_	456.71	Whole plant	Don et al. (2004)
225	2-Caffeoyloxy-3-hydroxy-3-(3,4-dihydroxyphenyl) propyl alcohol	C_18_H_18_O_8_	362.33	Whole plant	Don et al. (2004)
226	Octacosanol	C_28_H_58_O	410.76	Aerial part	Guo et al. (2005)
227	Triacontanoic acid	C_30_H_60_O_2_	452.81	Aerial part	Guo et al. (2005)

## 6 Biological activities

**FIGURE 2:**
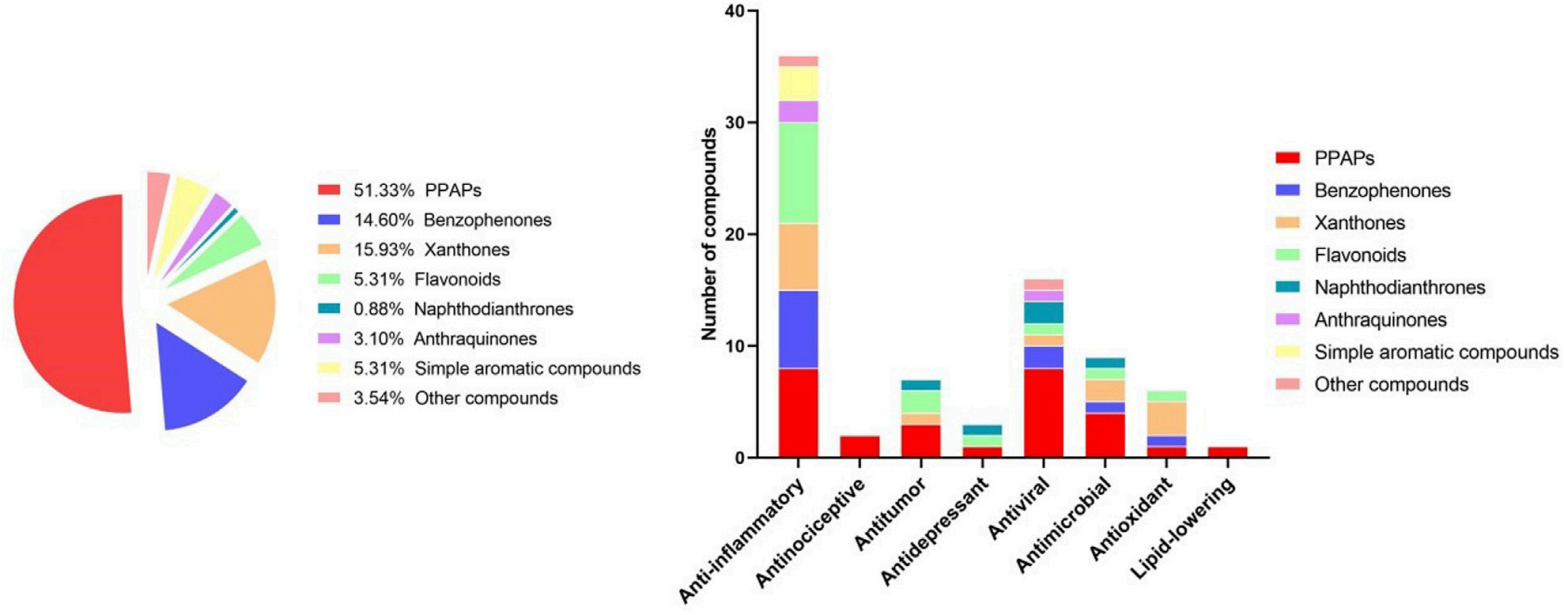
Graphical summary of the compounds and pharmacological activities of *H. sampsonii*.

Recent studies have revealed that several biological activities including anti-inflammatory, anti-tumor, anti-depressant, antiviral, antimicrobial, and antioxidant activities have been documented for extracts and secondary metabolites of *H. sampsonii* (Tian, 2015). These pharmacological effects have been summarized in Table 13; Figure 2.

**TABLE 13:** The biological activities of *H. sampsonii*.

Biological activities	Extracts/compounds	Models	Positive control	Results	Ref
Anti-inflammatory	Extracts	Dimethyl benzene-induced acute ear edema and carrageenin-induced paw oedema	Aspirin	Oedema↓	Pei et al. (2004)
	7-Epiclusianone (1)	Carrageenin-induced paw edema in rats and LPS-induced peritonitis in mice	Indomethacin	Paw oedema↓, leukocyte recruitment↓	Santa-Cecilia et al. (2011)
	Hyperforatin F (13)	BV-2, RAW 264.7, and THP-1 cells	Indomethacin	IC_50_ = 15.26 μΜ, 13.05 μΜ, and 18.05 μΜ, respectively	Chen et al. (2020)
	Hypericumone A (21)	RAW 264.7 cells	Andrographolide	IC_50_ = 40.32 μΜ	Huang et al. (2020)
	Norhypersampsone A (73)	RAW 264.7 cells	Quercetin	IC_50_ = 30.2 μM	Zhang et al. (2017)
	Otogirinin A (79)	RAW 264.7 cells	Andrographolide	IC_50_ = 32.87 μΜ	Huang et al. (2020)
	Sampsonione J (99)	RAW 264.7 cells	Andrographolide	IC_50_ = 35.25 μΜ	Huang et al. (2020)
	Sampsonol C (110)	RAW 264.7 cells	Indomethacin	IC_50_ = 27.3 μM	Xin et al. (2012)
	Sampsonol F (113)	RAW 264.7 cells	Indomethacin	IC_50_ = 29.3 μM	Xin et al. (2012)
	(E)-3-(3,7-dimethylocta-2,6-dienyl)2,4,6-trihydroxybenzophenone (121)	RAW 264.7 cells	Quercetin	IC_50_ = 37.1 μM	Zhang et al. (2017)
	(Z)-3-(3,7-dimethylocta-2,6-dienyl)2,4,6-trihydroxybenzophenone (122)	RAW 264.7 cells	Quercetin	IC_50_ = 36.5 μM	Zhang et al. (2017)
	4-geranyloxy-2,6-dihydroxybenzophenone (133)	RAW 264.7 cells	Quercetin	IC_50_ = 20.3 μM	Zhang et al. (2017)
	Garcimangosone D (136)	BV-2, RAW 264.7, and THP-1 cells	Indomethacin	IC_50_ = 14.52 μΜ, 17.23 μΜ, and 19.14 μΜ, respectively	Chen et al. (2020)
	Petiolin F (139)	RAW 264.7 cells	Cadamonin	IC_50_ = 2.00 μM	Dung Nguyen et al. (2021)
	Sampsine A (147)	RAW 264.7 cells	Cadamonin	IC_50_ = 2.40 μM	Dung Nguyen et al. (2021)
	Sampsine B (148)	RAW 264.7 cells	Cadamonin	IC_50_ = 2.29 μM	Dung Nguyen et al. (2021)
	1-hydroxy-7-methoxy-9H-xanthen-9-one (163)	BV-2, RAW 264.7, and THP-1 cells	Indomethacin	IC_50_ = 24.32 μΜ, 26.03 μΜ, 28.03 μΜ, respectively	Chen et al. (2020)
	2-methoxyxanthone (168)	BV-2, RAW 264.7, and THP-1 cells	Indomethacin	IC_50_ = 34.15 μΜ, 31.76 μΜ, and 37.64 μΜ, respectively	Chen et al. (2020)
	5′-Demethoxycadensin G (169)	BV-2, RAW 264.7, and THP-1 cells	Indomethacin	IC_50_ = 27.43 μΜ, 22.32 μΜ, and 37.64 μΜ, respectively	Chen et al. (2020)
	5-O-methyl-2-deprenyIrheediaxanthone B (171)	BV-2, RAW 264.7, and THP-1 cells	Indomethacin	IC_50_ = 19.96 μΜ, 18.92 μΜ, and 22.03 μΜ, respectively	Chen et al. (2020)
	Jacareubin (180)	BV-2, RAW 264.7, and THP-1 cells	Indomethacin	IC_50_ = 23.40 μΜ, 20.71 μΜ, and 26.65 μΜ, respectively	Chen et al. (2020)
	Mangiferin (181)	RAW 264.7, THP-1, AGS, NKE, MC3T3-E1, EA.hy926, ATDC5, 3T3-L1, primary mouse chondrocyte, human osteoarthritis chondrocyte, human renal glomerulus endothelial cell, and human oral epithelial cells; C57BL/6 mice, Balb/c mice, ICR mice, Swiss albino mice, Kunming mice, SD rats, and Wistar rats		Inhibited the expression of pro-inflammatory cytokines (TNF-α, IL-1β, IL-6) and COX-2, iNOS, IL-8, IRF5; regulated NF-κB, PI3K/AKT, and MAPK/ERK pathways	Mei et al. (2021)
	(+)-Catechin (187)	BV-2, RAW 264.7, and THP-1 cells	Indomethacin	IC_50_ = 25.31 μΜ, 26.29 μΜ, and 33.20 μΜ, respectively	Chen et al. (2020)
	3,8″-Biapigenin (188)	BV-2, RAW 264.7, and THP-1 cells	Indomethacin	IC_50_ = 21.15 μΜ, 19.05 μΜ, and 25.34 μΜ, respectively	Chen et al. (2020)
	Kaempferol (189)	BV-2, RAW 264.7, and THP-1 cells	Indomethacin	IC_50_ = 24.67 μΜ, 23.50 μΜ, and 29.57 μΜ, respectively	Chen et al. (2020)
	Naringenin (192)	BV-2, RAW 264.7, and THP-1 cells	Indomethacin	IC_50_ = 29.60 μΜ, 25.51 μΜ, and 31.16 μΜ, respectively	Chen et al. (2020)
	Quercetin (193)	BV-2, RAW 264.7, and THP-1 cells	Indomethacin	IC_50_ = 14.13 μΜ, 10.59 μΜ, and 15.92 μΜ, respectively	Chen et al. (2020)
	Hyperoside (194)	BV-2, RAW 264.7, and THP-1 cells	Indomethacin	IC_50_ = 24.84 μΜ, 21.70 μΜ, and 26.87 μΜ, respectively	Chen et al. (2020)
	Quercetin-3-O-arabinoside (197)	BV-2, RAW 264.7, and THP-1 cells	Indomethacin	IC_50_ = 40.32 μΜ, 31.82 μΜ, and 42.75 μΜ, respectively	Chen et al. (2020)
	Quercitrin (197)	BV-2, RAW 264.7, and THP-1 cells	Indomethacin	IC_50_ = 36.92 μΜ, 30.66 μΜ, and 38.71 μΜ, respectively	Chen et al. (2020)
	Rutin (198)	BV-2, RAW 264.7, and THP-1 cells	Indomethacin	IC_50_ = 32.35 μΜ, 27.17 μΜ, and 34.20 μΜ, respectively	Chen et al. (2020)
	3-ethyl-1,8-dihydroxy-6-methoxyanthracene-9, 10-dione (202)	BV-2, RAW 264.7, and THP-1 cells	Indomethacin	IC_50_ = 16.21 μΜ, 14.11 μΜ, and 17.90 μΜ, respectively	Chen et al. (2020)
	Emodin (203)	BV-2, RAW 264.7, and THP-1 cells	Indomethacin	IC_50_ = 17.06 μΜ, 12.39 μΜ, and 16.73 μΜ, respectively	Chen et al. (2020)
	3,4-dihydroxybenzoic acid (208)	BV-2, RAW 264.7, and THP-1 cells	Indomethacin	IC_50_ = 29.03 μΜ, 25.91 μΜ, and 30.25 μΜ, respectively	Chen et al. (2020)
	5,7-dihydroxy-3-methylchromone (211)	BV-2 and RAW 264.7 cells	Indomethacin	IC_50_ = 35.24 μΜ and 36.02 μΜ	Chen et al. (2020)
	Gallic acid (215)	BV-2, RAW 264.7, and THP-1 cells	Indomethacin	IC_50_ = 35.11 μΜ, 32.68 μΜ, and 38.42 μΜ, respectively	Chen et al. (2020)
	6-ethoxy-1H-pyrimidine-2,4-dione (220)	RAW 264.7 cells	Indomethacin	IC_50_ = 41.69 μΜ	Chen et al. (2020)
Antinociceptive	Extracts	Acetic acid writhing test and hot-plate test	Morphine	Writhing responses↓, pain thresold↑	Pei et al. (2004)
	7-Epiclusianone (1)	Acetic acid-induced writhing responses in mice, formalin test, hot plate test, and open-field test	Indomethacin, morphine	Writhing episodes↓, licking time↓, pain↓	Santa-Cecilia et al. (2011)
	Hyperforin (14)	Mice		Inhibited the activity of PKC	Galeotti et al. (2010)
Antitumor	Hyperforatin F (13)	SMMC-7721 cells	Cisplatin	IC_50_ = 10.00 μΜ	Guo et al. (2017)
	Hyperforin (14)	CLL, CML, AML, U937 cells and breast cancer cells		Induced apoptosis	Schiavone et al. (2014)
	Hyperisampsin J (32)	A549, HL-60, SMMC-7721, MCF-7, and SW480 cell lines		IC_50_ = 0.53 μΜ, 0.56 μΜ, 0.58 μΜ, 0.88 μΜ, 2.49 μΜ, respectively	Bridi et al. (2018)
	1,6-Dihydroxyxanthone (160)	Hela cells		IC_50_ = 33.00 μΜ	Wang et al. (2013)
	Quercetin (193)	U138MG, HeLa, U2-OS/MTX300, CWR22RV1, MDA-MB-453, HT-29, myeloid leukemia, and oral cavity cancer cell lines; CF1 mice, F344 rats, Wister rats, Min/+ mice, SD rats, CD-1 mice, and Swiss mice		Inhibited the proliferation of cancer cells; modulated the experimental carcinogenesis	Murakami et al. (2008), Dajas (2012)
	Rutin (198)	MDA-MB-231, HTC, HT29, A549, MCF-7, SW480 cell lines; HPV16 transgenic mice, HR-1 hairless mice		Induced the apoptosis in cancerous cells; COX2↓, inflammation↓	Imani et al. (2021)
	Hypericin (199)	U87 MG, U937, and K562 cells		Exhibited significant cytotoxic effects	Xu et al. (2015), Misuth et al. (2017)
Antidepressant	Hyperforin (14)	Forced swimming test in rats	Imipramine	Reduced the immobility time, inhibited the reuptake of neurotransmitters	Nahrstedt and Butterweck (2010), Bridi et al. (2018)
	Hyperoside (194)	Forced swimming test in rats	Imipramine	Reduced the immobility time	Nahrstedt and Butterweck (2010)
	Hypericin (199)	Forced swimming test in rats	Imipramine	Reduced the immobility time	Nahrstedt and Butterweck (2010)
Antiviral	Hyperisampsin A (23)	HIV		EC_50_ = 2.97 μM	Bridi et al. (2018)
	Hyperisampsin D (26)	HIV		EC_50_ = 0.97 μM	Bridi et al. (2018)
	Hypersampsone A (38)	HBV-producing cell line (MS-G2)		Isolated from the anti-HBV part	Lin and Wu (2003)
	Hypersampsone B (39)	HBV-producing cell line (MS-G2)		Isolated from the anti-HBV part	Lin and Wu (2003)
	Hypersampsone C (40)	HBV-producing cell line (MS-G2)		Isolated from the anti-HBV part	Lin and Wu (2003)
	Hypersampsone D (41)	HBV-producing cell line (MS-G2)		Isolated from the anti-HBV part	Lin and Wu (2003)
	Hypersampsone E (42)	HBV-producing cell line (MS-G2)		Isolated from the anti-HBV part	Lin and Wu (2003)
	Hypersampsone F (43)	HBV-producing cell line (MS-G2)		Isolated from the anti-HBV part	Lin and Wu (2003)
	2,6-Dihydroxy-4-[(E)-5-hydroxy-3,7-dimethylocta-2,7-dienyloxy]-benzophenone (128)	HBV-producing cell line (MS-G2)		Isolated from the anti-HBV part	Don et al. (2004)
	2,6-Dihydroxy-4-[(E)-7-hydroxy-3,7-dimethylocta-2-enyloxy]-benzophenone (129)	HBV-producing cell line (MS-G2)		Isolated from the anti-HBV part	Don et al. (2004)
	Hyperxanthone (178)	HBV-producing cell line (MS-G2)		Isolated from the anti-HBV part	Don et al. (2004)
	Kaempferol (189)	H5N1		Anti-H5N1	Yin (2014)
	Hypericin (199)	HSV, HCV, HIV, MCMV, Sindbis virus, infectious bronchitis virus, and novel duck reovirus		Exhibited significant inhibitory activity	Barnes et al. (2001), Zhang et al. (2022)
	Pseudohypericin (200)	HIV and HSV		Exhibited significant inhibitory activity	Barnes et al. (2001)
	R-(−)-skyrin-6-O-β-D-xylopyranoside (206)	HBV-producing cell line (MS-G2)		Isolated from the anti-HBV part	Don et al. (2004)
	2-caffeoyloxy-3-hydroxy-3-(3,4-dihydroxyphenyl) propyl alcohol (225)	HBV-producing cell line (MS-G2)		Isolated from the anti-HBV part	Don et al. (2004)
Antimicrobial	Root extract	MDR *S. aureus*	Norfloxacin	MIC = 64 μg/mL	Xiao et al. (2007)
	7-epiclusianone (1)	MDR *S. aureus*	Norfloxacin	MIC = 4 μg/mL	Xiao et al. (2007)
	Hyperforin (14)	MRSA and PRSA		MIC <6 μM; MIC = 0.1–1.0 μg/mL	Schiavone et al. (2014), Bridi et al. (2018)
	Peroxysampsone A (84)	*S. aureus*	Norfloxacin	Exhibited Comparable activity with the positive drug	Xiao et al. (2010)
	Sampsone A (89)	MRSA		MIC = 32 μg/mL	Xin et al. (2011b)
	4-geranyloxy-2,6-dihydroxybenzophenone (133)	*Klebsiella pneumoniae*, *Mycobacterium smegmatis*, *Pseudomonas aeruginosa*, *Salmonella gallinarum*, and *S. aureus*		Exhibited inhibitory activity	Pecchio et al. (2006)
	Hypericumxanthone A (174)	MRSA		MIC = 16 μg/mL	Xin et al. (2011b)
	Hypericumxanthone B (175)	MRSA		MIC = 32 μg/mL	Xin et al. (2011a)
	Quercetin (193)	*S*. *aureus, Pseudomonas aeruginosa, Streptococcus mutans*, *Streptococcus sobrinus*, *Lactobacillus acidophilu*, *Streptococcus sanguis*, *Actinobacillus actinomycetemocomitans,* and *Prevotella intermedia*		Exhibited inhibitory activity	Nguyen and Bhattacharya (2022)
	Hypericin (199)	*S. aureus*		Stopped the growth of *S. aureus* when incubated with 40 μM combined with visible light	Wölfle et al. (2014)
Antioxidant	Ethyl acetate extract	BALB/c mice	5-ASA	Regulated the levels of CAT, GSH, MDA, and SOD	Lin et al. (2022)
	Hyperforin (14)	HaCaT cell line	Trolox and Nacetylcysteine	EC_50_ = 0.42 μg/mL, higher than Trolox (12 μg/mL) and Nacetylcysteine (847 μg/mL)	Wölfle et al. (2014)
	Mangiferin (181)	DPPH assay		IC_50_ = 35.48 μM	Dung Nguyen et al. (2021)
	Kaempferol (189)	DPPH assay		Showed 66% scavenging activity at the concentration of 75 μM	Deng et al. (2019)
	Quercetin (193)	DPPH assay		Inhibition of MAO	Stojanović et al. (2013)
	Rutin (198)	SH-SY5Y cell line		Increased the production of SOD, CAT and GSH	Enogieru et al. (2021)
	Hypericin (199)	MCF-7 cell line		Increased the production of SOD-2 combined with photodynamic therapy	Kimáková et al. (2017)
Lipid-lowering	Hypersampone A (114)	HepG2 cell line	Rosiglitazone	Inhibited the expression of FAS and ACACA at 5 μM	Huang et al. (2022)

### 6.1 Anti-inflammatory activity

*In vitro* studies have suggested that extracts of *H. sampsonii* showed anti-inflammatory activity in lipopolysaccharide (LPS)-treated BV-2, RAW 264.7, and THP-1 cells (Chen et al., 2020). On the other hand, *in vivo* studies have demonstrated that the alcohol extracts of *H. sampsonii* had antinociceptive and anti-inflammatory properties. The antinociceptive potential carried out using acetic acid-induced writhing responses in mice and hot-plate test suggested that extracts of *H. sampsonii* effectively suppressed the writhing symptom and increased the pain threshold of mice. Also, the anti-inflammatory effect was investigated using dimethyl benzene-induced acute ear edema and carrageenin-induced paw edema in rats. Besides, the anti-inflammatory activity have been demonstrated by the reduction of acute ear edema induced by dimethyl benzene and carrageenin-induced paw oedema (Pei et al., 2004). A study of our group to investigate the therapeutic effects and molecular mechanisms of *H. sampsonii* (HS) in a dextran sulfate sodium (DSS)-induced ulcerative colitis (UC) mice model (Lin et al., 2022). These results indicate that HS distinctly alleviated DSS-stimulated UC-like lesions symptoms as evidenced by a significant recovery from body weight, colon lengths, and histological injuries of colons. HS reduced the accumulation of pro-inflammatory cytokines and improved the mRNA level of IL-10. Simultaneously, the colonic mRNA expression levels of IL- 1β, IL-17, iNOS and COX-2 were all significantly suppressed by HS in a dose-dependent manner. Furthermore, HS restored the protein expression of tight junction-associated protein (ZO-1 and occluding). Further studies have also reported that HS can significantly inhibit the protein level of PDE4 and reduced the expressions of PKA and phosphorylated CREB.

Experimental evidence has emerged to indicate that PPAPs are one of the major constituents required for anti-inflammatory effects. Since, it has been reported that a series of compounds isolated from *H. sampsonii*, including hyperattenin C (**3**), otogirinin D (**12**), hyperisampsin I (**15**), hyperisampsin J (**16**), sampsonione L (**28**), hyperattenin G (**38**), hypersampsone O (**63**), hypersampsonone A (**68**), sampsonione A (**85**), and sampsonione B (**86**), were found to have significant PDE4D2 inhibitory activity (Zhang et al., 2016). PDE4D2 is one of the subtypes of phosphodiesterase-4 (PDE4), which can specifically hydrolyze cAMP and participate in various physiological responses, and is a promising drug target for inflammatory diseases such as psoriasis and ulcerative colitis. Moreover, the antinociceptive and anti-inflammatory properties have been reported for 7-epiclusianone (**1**) using animal models (Santa-Cecilia et al., 2011). In addition to PPAPs, other compounds such as benzophenone, xanthones, flavonoids, anthraquinones, and phenols, are also stated to possess anti-inflammatory properties (Chen et al., 2020).

Besides, benzophenone derivatives have also been shown to be important in the anti-inflammatory effects of *H. sampsonii*. Recently, our group investigated the therapeutic effect and potential mechanisms of 4-geranyloxy-2,6-dihydroxybenzophenonel (4-GDB, **133**) on DSS-induced ulcerative colitis in mice (Wang et al., 2023). This study showed that intragastric administration of 4-GDB (20 mg/kg/day) for 8 days significantly attenuated colonic injury, reduced the expression of inflammatory mediators, and improved colonic barrier function in mice with colitis. Furthermore, *in vivo* and *in vitro* experiments indicated that 4-GDB could activate cAMP/PKA/CREB and inhibit the NF-κB pathway. Collectively, 4-GDB may be a potential agent for treating UC by regulating the cAMP/PKA/CREB and NF-κB pathways.

### 6.2 Antitumor activity

The antitumor activity of *H. sampsonii* has been evaluated in various cancer cell lines *in vitro* including A375, MDA-MB-231, SHSY-5Y, and SiHa cell lines (Chen et al., 2020). Studies have suggested that regulation of subcellular localization of retinoid X receptor-alpha (RXR-α) is a potential method to induce tumor cell apoptosis. Zeng et al. have found that *H. sampsonii* extracts can induce the translocation of RXR-α from the nucleus to the cytoplasm, and promote the apoptosis of NIH-H460, MGC-803, and SMMC-7721 (Jin-Zhang et al., 2006). Besides, the ethanol extract and the chloroform fraction especially were demonstrated for apoptosis-inducing and antitumor properties via inhibiting RXR-α transcription (Han et al., 2007).

Moreover, the antitumor effect has also been documented for a panel of natural products in *H. sampsonii*, such as 7-epiclusianone (**1**) (Sales et al., 2015), sampsonione A (**85**) (Hu and Sim, 1999a), sampsonione I (**93**) (Hu and Sim, 1999a), mangiferin (**181**) (Mei et al., 2021), naringenin (**192**) (Memariani et al., 2021), quercetin (**193**) (Murakami et al., 2008; Dajas, 2012) and rutin (**198**) (Imani et al., 2021).

### 6.3 Antidepressant activity

Depression is a common mental disorder characterized by syndromes like depressed mood, hopelessness, and even thoughts of suicide. Clinical studies have demonstrated that *H. perforatum* L. (St. John’s Wort), a member of the Hypericum genus, has significant antidepressant impacts. The extract of this plant was introduced into the market as an antidepressant in Germany in 1988, and became the preferred phytomedicine for the treatment of depressive disorder in European and American regions. Intriguingly, previously reported studies have also isloated an antidepressant active metabolite hyperforin rom *H. perforatum* and its also present in *H. sampsonii* (Zheng et al., 2003).

In 2003, the ethanol extracts of *H. sampsonii* were demonstrated for a significant antidepressant effect on the behavior despair animal models (Wan et al., 2003). It was believed that the total flavonoids of *H. sampsonii* showed antidepressant activity in the hypothermia experiments induced by reserpine and the forced swimming test. In studies utilizing the forced swimming test, tail suspension test, and open-field test, *H. sampsonii* extracts, HTX fraction, and mangiferin (**181**) induced a significant reduction in immobility, and the antidepressant mechanism of HTX might be related to neurotransmitters (Gong, 2014). The antidepressant properties of *H. sampsonii* have been attributed to various phytochemical constituents, such as hyperforin (**6**), hyperoside (**194**), and hypericin (**199**) (Nahrstedt and Butterweck, 2010; Bridi et al., 2018). However, the precise mechanism of action for the antidepressant capacity of this plant remains indistinct.

### 6.4 Antiviral activity

Previous studies have suggested that the extracts and several compounds of *H. sampsonii* have antiviral activity. For instance, the chloroform and n-butyl alcohol fractions as well as kaempferol (**189**) were shown to possess antiviral activity against avian influenza virus H5N1 in the Madin Darby Canine Kidney (MDCK) screening experiment (Yin, 2014). Besides, Lin and Wu found that hypersampsone A-F (**52–56**, **9**) isolated from *H. sampsonii* exhibited anti-HBV activity on the MS-G2 cell line (Lin and Wu, 2003). In addition, the antiviral activity has been reported for hypericin (**199**) and pseudohypericin (**200**) against herpes simplex virus types 1 and 2 and HIV-1 *in vitro*. Hypericin (**199**) has also exhibited activity against HCV, murine cytomegalovirus (MCMV), Sindbis virus, infectious bronchitis virus, and novel duck reovirus (Barnes et al., 2001; Zhang et al., 2022).

### 6.5 Antimicrobial activity

Plants belonging to the Hypericum genus are a crucial source of antimicrobial compounds (Marrelli et al., 2016). Previous evidence indicated that the antibacterial activity has been demonstrated for hyperforin (**6**) and quercetin (**193**) against *Staphylococcus aureus*, *Streptococcus mutans*, *Streptococcus pyogenes*, and *Corynebacterium diphtheria*, etc (Barnes et al., 2001; Nguyen and Bhattacharya, 2022). In studies using MDR *S. aureus* strain SA-1199B to determine the antibacterial effect of *H. sampsonii*, the MIC of the petroleum ether extract of the root was up to 64 μg/mL (Xiao et al., 2007). Moreover, 7-epiclusianone (**1**) induced potent antibacterial activity against SA-1199B with a MIC of 4 μg/mL, while MIC of the positive control (norfloxacin) was 32 μg/mL (Xiao et al., 2007). In other antibacterial experiments, some PPAPs including sampsone A (**107**) and hypericumxanthone A (**174**) were shown to exhibit good antibacterial activity on Methicillin-resistant *S*. *aureus* (MRSA), with MIC values of 32 μg/mL and 16 μg/mL respectively (Xin et al., 2011).

### 6.6 Antioxidant activity

Reactive oxygen species (ROS), the important substances released from neutrophils, play a part in cell signaling and homeostasis. It is, however, important to note, that the overproduction of ROS can initiate the inflammatory cascade and subsequent cell damage as well as tissue dysfunction under oxidative stress (Chen et al., 2009). Research revealed that *H. sampsonii* showed antioxidant capacity by regulating the content of oxidase (GSH and SOD) (Chen et al., 2009). It has also been reported that the ethyl acetate extract of *H. sampsonii* could alleviate oxidative stress as indicated by reversing the abnormal levels of CAT, GSH, MDA, and SOD in mice with colitis (Lin et al., 2022). Additionally, the antioxidant activity of mangiferin (**181**) from *H. sampsonii* was assessed by means of the DPPH radical scavenging assay with an IC_50_ value of 35.48 μM (Dung Nguyen et al., 2021).

## 7 Safety

Many ancient classics and medicinal books have recorded that the clinical administration dosage of *H. sampsonii* should be 9–15 g for dried herb or 30–60 g for fresh herb. To further determine the safety of therapeutic doses of *H. sampsonii*, a previous study by Lin et al. (2022), fed mice with the ethyl acetate extract at a dose of 2000 mg/kg. After 14 days of observation, there was no morphological abnormality in major organs, indicating that the ethyl acetate extract of *H. sampsonii* showed no toxicity. Although the toxicity studies and the wide range of edible and medicinal values of *H. sampsonii* may provide a preliminary reference for its high safety in clinical application; however, the potential toxicity cannot be completely excluded.

According to the *Chinese Materia Medica*, morphological and microscopic examinations as well as physicochemical identification should be used to control the quality of *H. sampsonii*. Meanwhile, for its medicinal application, it is should also contain hypericin (**199**) and flavonoids (Editorial Board of Chinese Materia Medica, 1999). Yet, thin-layer chromatography (TLC) identification and content determination as well as other analytical methods have not been employed to control the quality of this plant, indicating a lack of quality standard despite its extensive folk utilization. Besides, there is no indication of potential safety issues. Therefore, further research work is essentially required to meet these standards.

## 8 Conclusion and perspectives

As a common botanical drug for the treatment of dysentery, enteritis, and irregular menstruation in folk, *H*. *sampsonii* is safe and effective. It is a versatile plant with a complexity of phytochemicals and remarkable pharmacological actions. In this paper, we reviewed the botany, traditional uses, phytochemistry, pharmacological activities, and safety of this species for the first time. It was found that more than 220 chemicals have been isolated and identified from this plant, including PPAPs, benzophenones, xanthones, flavonoids, naphthodianthrones, anthraquinones, and aromatic compounds, among others. Among the identified compounds, PPAPs are the most abundant compounds with novel structures as well as up-and-coming biological characteristics, such as PDE4 inhibitory activity. Although, accumulating studies have shown the progress in the understanding of its anti-inflammatory, anti-tumor, antidepressant, antiviral, antibacterial, and antioxidant properties. However, further studies should focused on the isolation of new compounds and biological screening tests *in vitro* from *H. sampsonii.* Yet, it is also important to mention that empirical pharmacologic studies are insufficient to validate the claimed healing properties.

It is noteworthy that *H. perforatum* L., the most familiar species of the Hypericum genus, has been extensively investigated due to its medicinal values and was listed in the Chinese Pharmacopoeia in 2015. Nevertheless, *H*. *sampsonii* has not yet been listed in the Chinese Pharmacopoeia, which potentially prevents in-depth research to a large extent. Taken together, the current pharmacological research on *H*. *sampsonii* remains in infancy, and other aspects such as safety evaluation and quality control standards are scanty. This review provided a systematic overview of this plant based on the available research while not comprehensive. Therefore, further studies including pharmacological mechanisms *in vitro* and *in vivo*, structure-activity relationship appraisal, safety evaluation, and quality standards should be done. More emerging studies may reveal the scientific connotation of the traditional application and lay the foundation for the development and utilization of *H*. *sampsonii*.

## Abbreviations

CNKI, China National Knowledge Infrastructure; IC_50_, 50% Inhibitory concentration; uM, Micromolar; Pre, isoprenyl; Ger, geranyl; Bz, benzoyl.
