# Supplementary material for: Hypericum sampsonii Hance: a review of its botany, traditional uses, phytochemistry, biological activity, and safety
Source: Front Pharmacol. 2023 Sep 19;14:1247675. doi: 10.3389/fphar.2023.1247675 (PMC10546196; doi:10.3389/fphar.2023.1247675)
Supplement: Supplementary file 1 [file Presentation1.zip › Supplementary material.docx]

Supplementary material

S1. Structures of BPAPs (1–32) isolated from H. sampsonii.

S2. Structures of Caged PPAPs (33–96) isolated from H. sampsonii.

S3. Structures of other PPAPs (97–116) isolated from H. sampsonii.

S4. Structures of benzophenones (117–150) isolated from H. sampsonii.

S5. Structures of xanthones (151–186) isolated from H. sampsonii.

S6. Structures of flavonoids (187–198) isolated from H. sampsonii.

S7. Structures of naphthodianthrones (199–200) isolated from H. sampsonii.

S8. Structures of anthraquinones (201–207) isolated from H. sampsonii.

S9. Structures of simple aromatic compounds (208–219) isolated from H. sampsonii.

S10. Structures of other compounds (220–227) isolated from H. sampsonii.


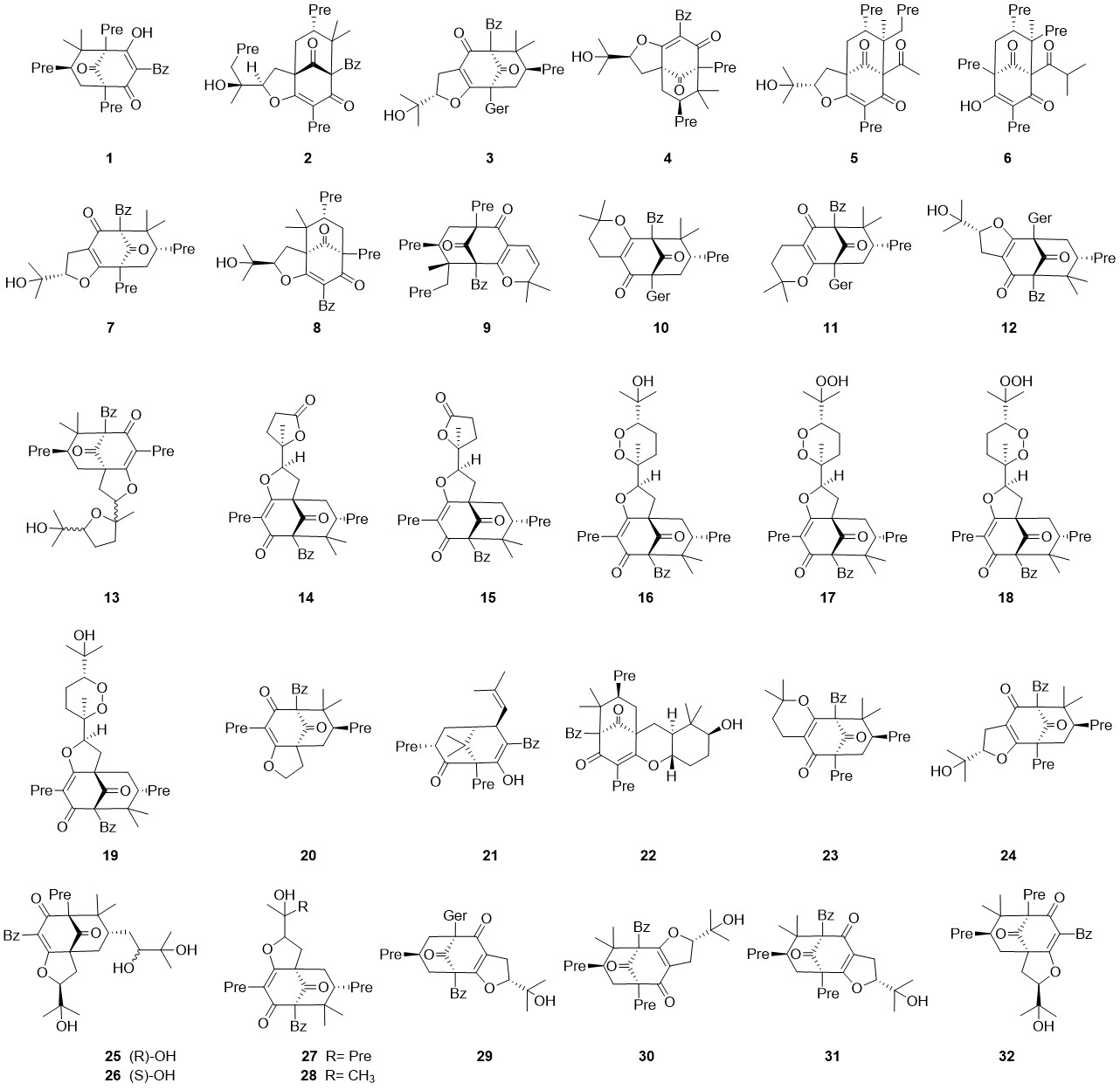


S1. Structures of BPAPs (1–32) isolated from H. sampsonii.


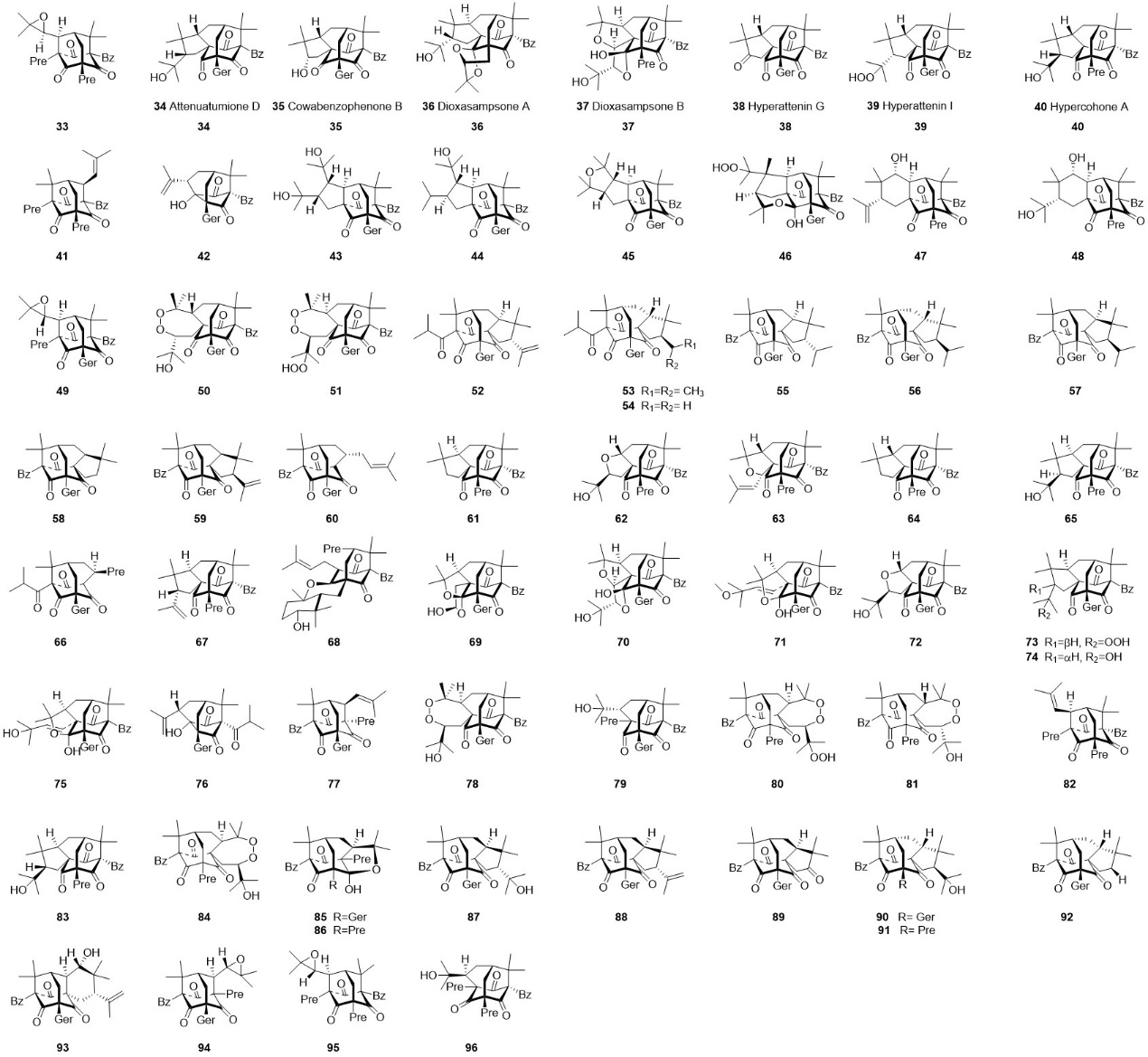


S2. Structures of Caged PPAPs (33–96) isolated from H. sampsonii.


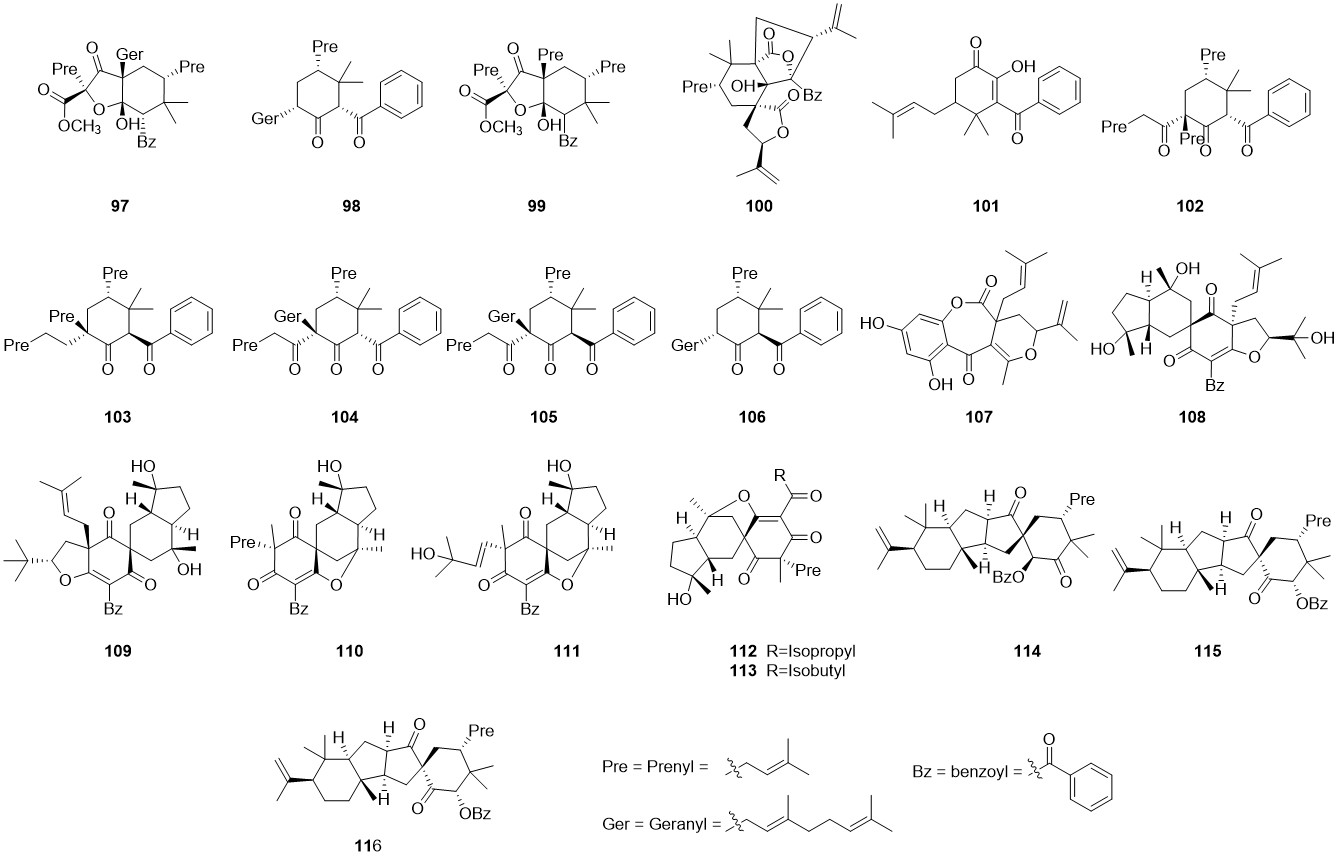


S3. Structures of other PPAPs (97–116) isolated from H. sampsonii.


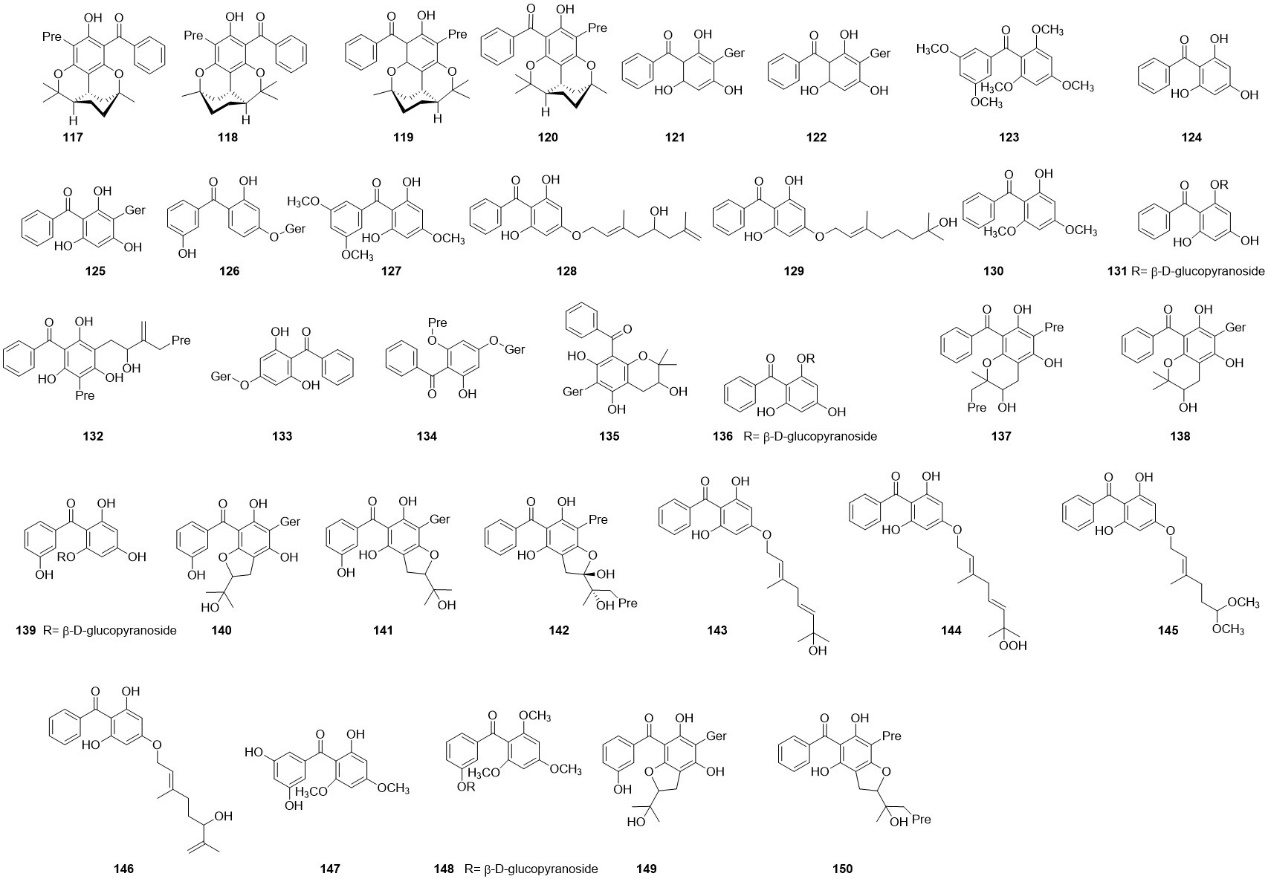


S4. Structures of benzophenones (117–150) isolated from H. sampsonii.


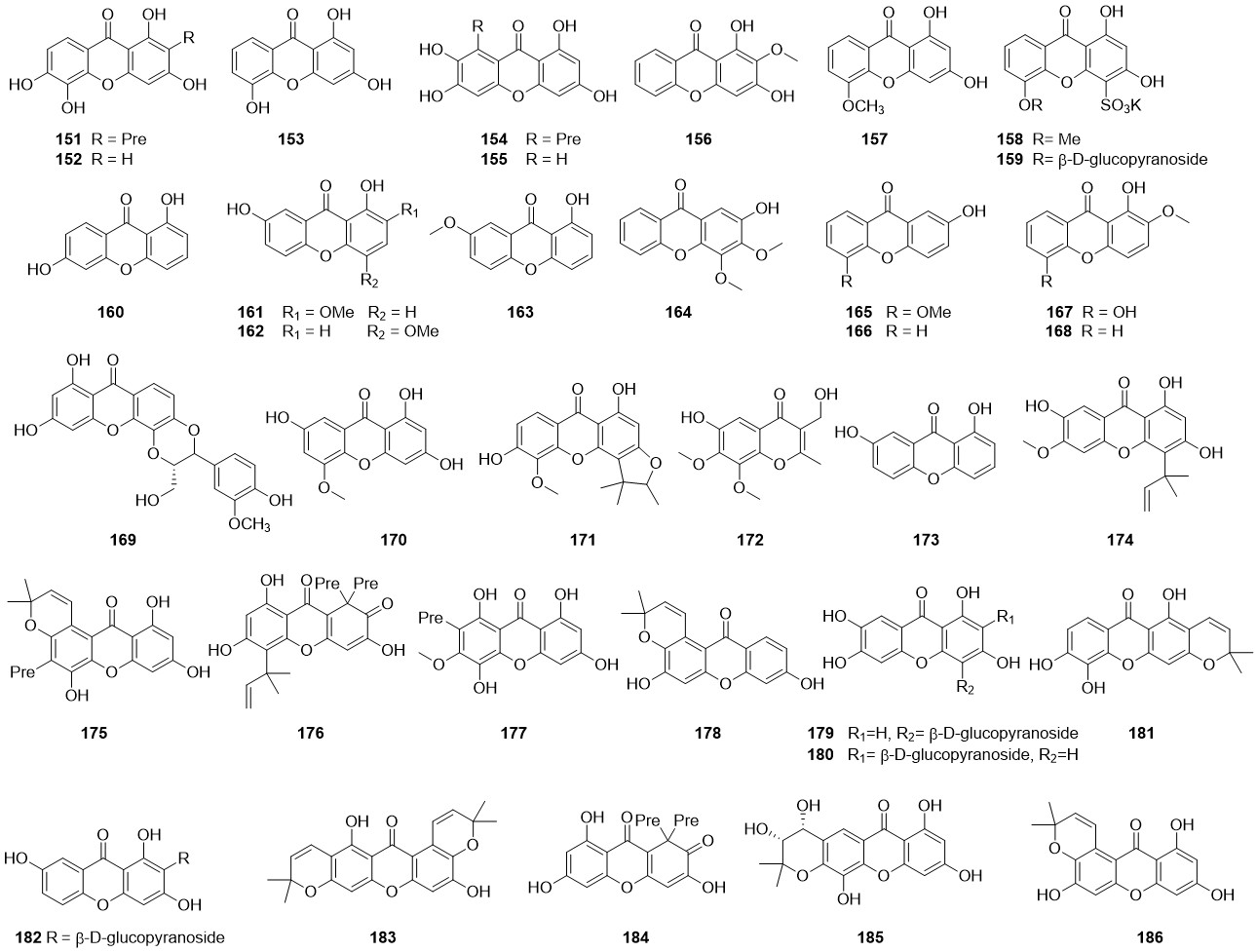


S5. Structures of xanthones (151–186) isolated from H. sampsonii.


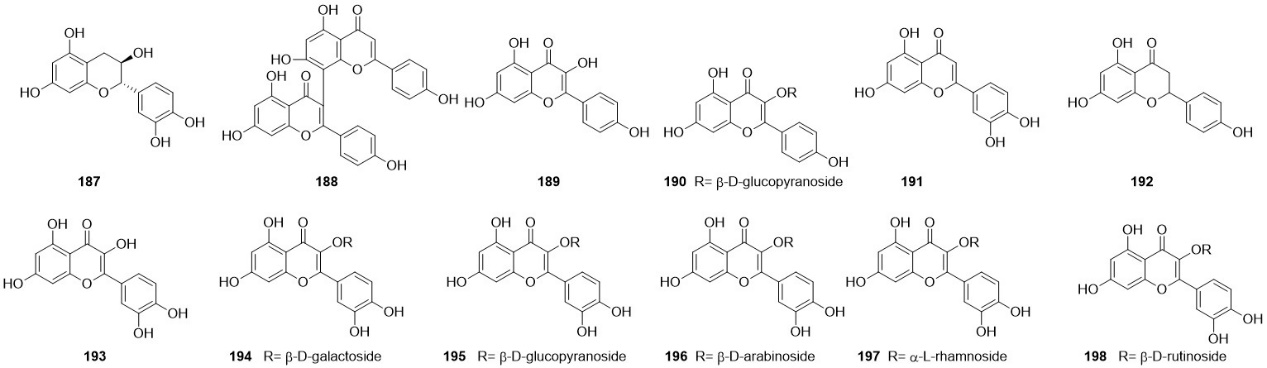


S6. Structures of flavonoids (187–198) isolated from H. sampsonii.


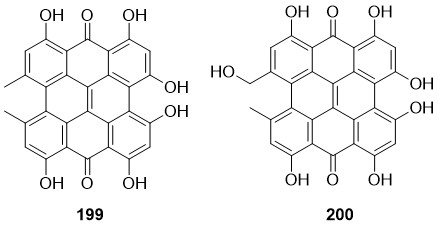


S7. Structures of naphthodianthrones (199–200) isolated from H. sampsonii.


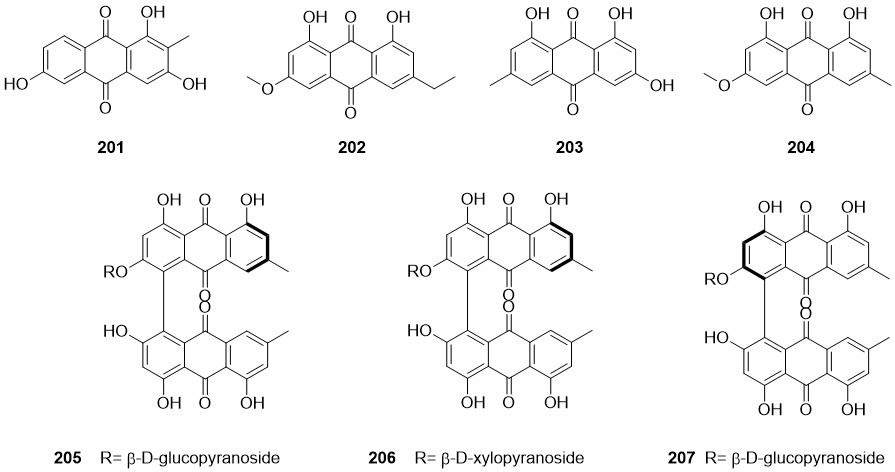


S8. Structures of anthraquinones (201–207) isolated from H. sampsonii.


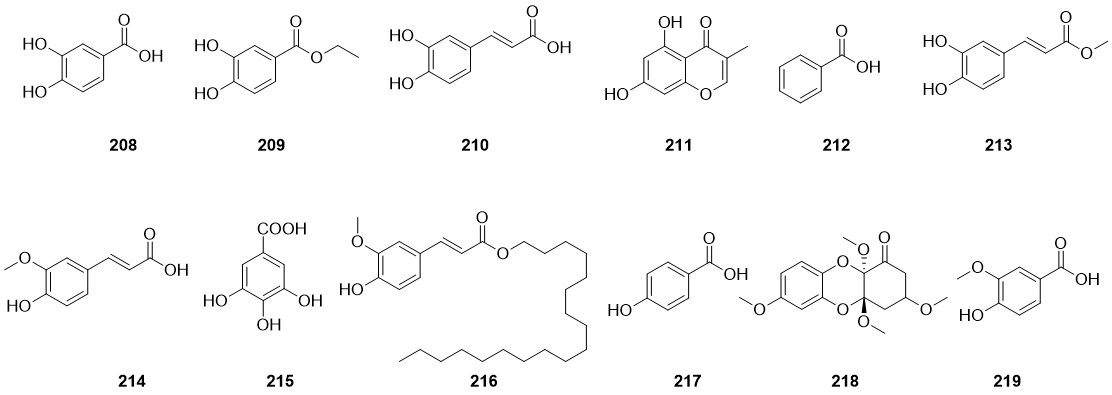


S9. Structures of simple aromatic compounds (208–219) isolated from H. sampsonii.


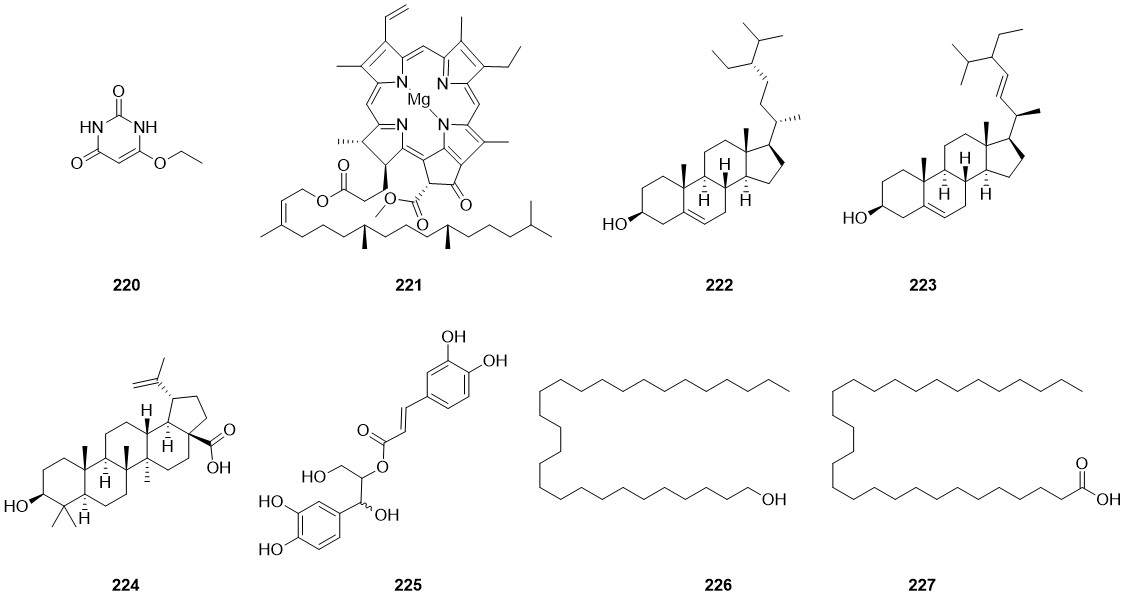


S10. Structures of other compounds (220–227) isolated from H. sampsonii.
