# Supplementary figures and images for: Hypericum sampsonii Hance: a review of its botany, traditional uses, phytochemistry, biological activity, and safety
Source: Front Pharmacol. 2023 Sep 19;14:1247675. doi: 10.3389/fphar.2023.1247675 (PMC10546196; doi:10.3389/fphar.2023.1247675)

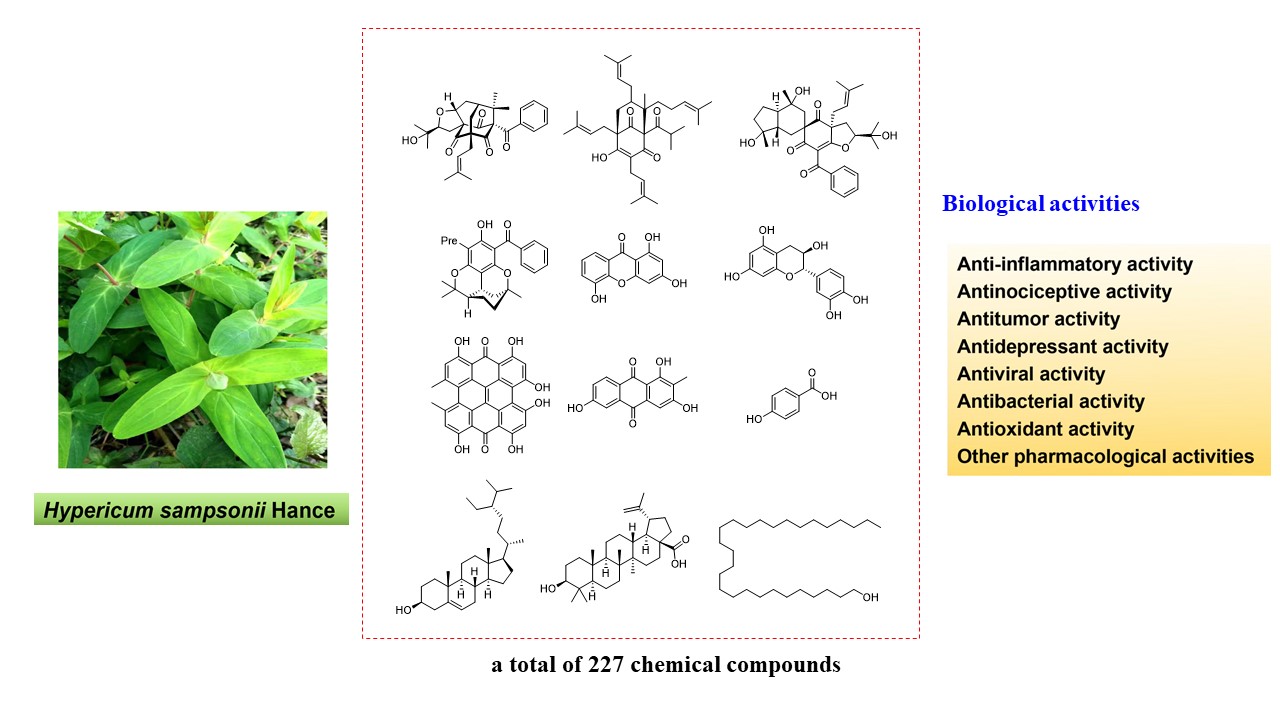

Supplement: Supplementary file 2 [file Image1.JPEG]
